# The superantigens SpeC and TSST-1 specifically activate TRBV12-3/12-4^+^ memory T cells

**DOI:** 10.1038/s42003-023-04420-1

**Published:** 2023-01-20

**Authors:** Freya R. Shepherd, Kate Davies, Kelly L. Miners, Sian Llewellyn-Lacey, Simon Kollnberger, James E. Redman, Melissa M. Grant, Kristin Ladell, David A. Price, James E. McLaren

**Affiliations:** 1 Division of Infection and Immunity, School of Medicine, Cardiff University, Cardiff, UK; 2 School of Chemistry, Cardiff University, Cardiff, UK; 3 School of Dentistry, Institute of Clinical Sciences, University of Birmingham, Birmingham, UK; 4 Systems Immunity Research Institute, School of Medicine, Cardiff University, Cardiff, UK

**Keywords:** Bacterial infection, Immunological memory

## Abstract

Severe bacterial or viral infections can induce a state of immune hyperactivation that can culminate in a potentially lethal cytokine storm. The classic example is toxic shock syndrome, a life-threatening complication of *Staphylococcus aureus* or *Streptococcus pyogenes* infection, which is driven by potent toxins known as superantigens (SAgs). SAgs are thought to promote immune evasion via the promiscuous activation of T cells, which subsequently become hyporesponsive, and act by cross-linking major histocompatibility complex class II molecules on antigen-presenting cells to particular β-chain variable (TRBV) regions of αβ T cell receptors (TCRs). Although some of these interactions have been defined previously, our knowledge of SAg-responsive TRBV regions is incomplete. In this study, we found that CD4^+^ and CD8^+^ T cells expressing TRBV12-3/12-4^+^ TCRs were highly responsive to streptococcal pyrogenic exotoxin C (SpeC) and toxic shock syndrome toxin-1 (TSST-1). In particular, SpeC and TSST-1 specifically induced effector cytokine production and the upregulation of multiple coinhibitory receptors among TRBV12-3/12-4^+^ CD4^+^ and CD8^+^ memory T cells, and importantly, these biological responses were dependent on human leukocyte antigen (HLA)-DR. Collectively, these data provided evidence of functionally determinative and therapeutically relevant interactions between SpeC and TSST-1 and CD4^+^ and CD8^+^ memory T cells expressing TRBV12-3/12-4^+^ TCRs, mediated via HLA-DR.

## Abstract (web summary)

In peripheral blood mononuclear cells from healthy human donors, the superantigens SpeC and TSST-1 are shown to specifically activate TRBV12-3/12-4^+^ memory T cells.

## Introduction

The cellular immune response to infection typically involves a complex but coordinated biological network that has evolved to limit microbial spread and restore homeostasis in the absence of pathology. However, severe infections can overwhelm the immune system, leading to generalized hyperinflammation and the induction of a potentially lethal cytokine storm. These adverse outcomes are typically associated with bacterial pathogens, such as *Staphylococcus aureus* (*S. aureus*), but can also complicate viral infections, as exemplified recently in the context of SARS-CoV-2^1^.

Toxic shock syndrome is a life-threatening complication of infection with *S. aureus* or *Streptococcus pyogenes* (*S. pyogenes*) driven by potent toxins known as superantigens (SAgs), which likely emerged to facilitate immune evasion^2,3^ and promote bacterial colonization^4^. SAgs disrupt adaptive immune responses by hyperactivating CD4^+^ and CD8^+^ T cells^5,6^, which subsequently become anergic or hyporesponsive^7^, in part due to the upregulation of various coinhibitory receptors (coIRs)^8^. These effects are induced mechanistically via direct cross-linking of major histocompatibility complex (MHC) class II molecules on the surface of antigen-presenting cells to T cell receptor (TCR) β-chain variable (TRBV) regions of the clonotypically expressed αβ TCR^6,9–13^, thereby circumventing the requirement for peptide specificity^14,15^. SAgs can also bind some TCR α-chains, as exemplified by staphylococcal enterotoxin H (SEH)^13^, and costimulatory molecules^16–18^.

SAgs can engage multiple TRBV regions^5^, although some appear to be highly selective, including toxic shock syndrome toxin-1 (TSST-1), which interacts with TRBV20-1^10,19^. However, *S. aureus* encodes 26 known SAgs, including 11 distinct enterotoxins and TSST-1, and *S. pyogenes* encodes 14 known SAgs, including streptococcal pyrogenic exotoxin A (SpeA) and SpeC^5^. Such collective diversity overcomes individual specificity limitations and allows these bacteria to target many different TCRs^5,20^, although currently, some TRBV regions have not been mapped to defined SAgs^5^. This potential knowledge gap is important, because SAgs have been strongly linked with many life-threatening conditions, including pneumonia, infective endocarditis, sepsis, and Kawasaki-like disease^20,21^. In addition, modified forms of these toxins have been used as vaccines^22^, and molecular antagonists have been developed to inhibit the biological effects of SEB^23^.

Human T cell responses against many viruses, including cytomegalovirus (CMV) and human immunodeficiency virus (HIV), can be driven by “public” T cell clonotypes, which recur in multiple individuals and express identical TCRs^24–27^. Repertoire bias is also common. For example, CD8^+^ T cell responses directed against specific epitopes from HIV^28^ and dengue virus^29^, each restricted by a distinct human leukocyte antigen (HLA) class I molecule, often incorporate TRBV12-3/12-4^+^ TCRs. More generally, biased TRBV use is a particular feature of unconventional antigen recognition, typified by mucosal-associated invariant T (MAIT) cells^30^. These conserved patterns of repertoire deployment likely drove the evolution of particular SAgs to disrupt specific immune responses against bacteria^8^ and viruses^31,32^. In addition, SAgs can induce TCR downregulation and/or internalization in a TRBV-specific manner, negating antigen recognition completely^33^.

To extend the current knowledge base, which has yet to encompass all potentially relevant interactions, we mapped a range of SAgs onto the landscape of functionally responsive TCRs. In addition to previously reported specificities, we found that TRBV12-3/12-4^+^ CD4^+^ and CD8^+^ T cells responded vigorously to SpeC and TSST-1. The induced responses were functionally replete and associated with the upregulation of multiple coIRs. Moreover, activation occurred in the absence of SAg-induced downregulation and/or internalization of the corresponding TCRs, suggesting a distinct mechanism of action that was nonetheless dependent on HLA-DR.

## Results

### SpeC and TSST-1 activate TRBV12-3/12-4^+^ CD4^+^ and CD8^+^ T cells

**Fig. 1:**
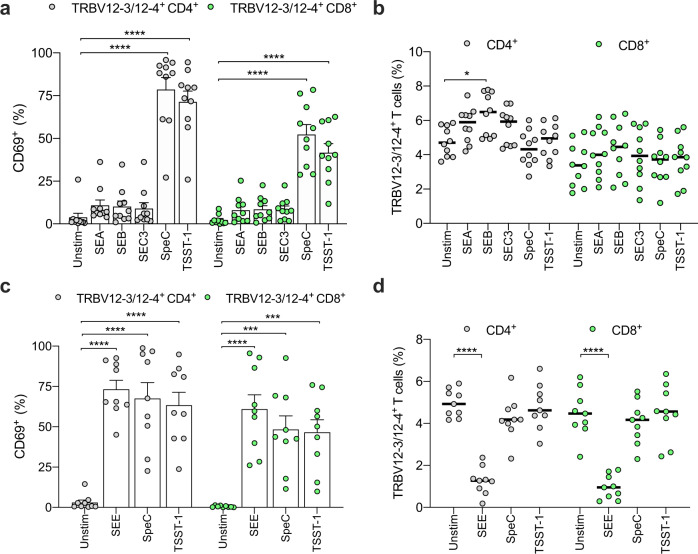
SpeC and TSST-1 activate TRBV12-3/12-4^+^ CD4^+^ and CD8^+^ T cells. **a** Frequency of CD69^+^ cells among TRBV12-3/12-4^+^ CD4^+^ (gray filled circles) or CD8^+^ T cells (green filled circles) from human PBMCs cultured in medium alone (unstim) or stimulated for 24 h with SEA, SEB, SEC3, SpeC, or TSST-1. Each dot represents one donor. Data are shown as mean ± SEM. ****p < 0.0001. One-way ANOVA with Tukey’s post-hoc test. **b** Frequency of TRBV12-3/12-4^+^ cells among CD4^+^ (gray filled circles) or CD8^+^ T cells (green filled circles) from human PBMCs cultured in medium alone (unstim) or stimulated for 24 h with SEA, SEB, SEC3, SpeC, or TSST-1. Each dot represents one donor. Horizontal bars indicate median values. *p < 0.05. One-way ANOVA using transformed data with Tukey’s post-hoc test. **c** Frequency of CD69^+^ cells among TRBV12-3/12-4^+^ CD4^+^ (gray filled circles) or CD8^+^ T cells (green filled circles) from human PBMCs cultured in medium alone (unstim) or stimulated for 24 h with SEE, SpeC, or TSST-1. Each dot represents one donor. Data are shown as mean ± SEM. ***p < 0.001, ****p < 0.0001. One-way ANOVA with Tukey’s post-hoc test. **d** Frequency of TRBV12-3/12-4^+^ cells among CD4^+^ (gray filled circles) or CD8^+^ T cells (green filled circles) from human PBMCs cultured in medium alone (unstim) or stimulated for 24 h with SEE, SpeC, or TSST-1. Each dot represents one donor. Horizontal bars indicate median values. ****p < 0.0001. One-way ANOVA using transformed data with Tukey’s post-hoc test.

In preliminary experiments, we used a panel of 16 TCR Vβ-specific antibodies to investigate SAg response patterns among CD4^+^ and CD8^+^ T cells, measuring activation via the upregulation of CD69. Peripheral blood mononuclear cells (PBMCs) were isolated from healthy donors and stimulated for 24 h with commercially available recombinant forms of SEA, SEB, SEC3, or TSST-1 from *S. aureus* or SpeC from *S. pyogenes*. The gating strategy is shown in Fig. S1. Earlier findings were confirmed using this approach. In particular, TCR Vβ1^+^ (TRBV9^+^) cells were activated by SEA and SEB, whereas TCR Vβ12^+^ (TRBV10-1/10-2/10-3^+^) cells were activated by SEB and SEC3 (Fig. S2a)^33–35^, and TCR Vβ2^+^ (TRBV20-1^+^) cells were activated by SpeC and TSST-1 (Fig. S2b)^12,19,36^. We also found that TCR Vβ8^+^ (TRBV12-3/12-4^+^) cells were highly responsive to SpeC and TSST-1 but not to SEA, SEB, or SEC3 (Fig. 1a). These observations were validated using another marker of cellular activation, namely CD25 (Fig. S2c).

TSST-1 is known to downregulate TRBV20-1^+^ TCRs^33^. To extend this finding, we measured the frequencies of various TRBV-defined CD4^+^ and CD8^+^ T cells among PBMCs before and after stimulation with SpeC or TSST-1. Unexpectedly, the frequencies of TRBV12-3/12-4^+^ CD4^+^ and CD8^+^ T cells remained largely unchanged after exposure to these SAgs (Fig. 1b), whereas the frequencies of TRBV20-1^+^ CD4^+^ and CD8^+^ T cells were significantly reduced by SpeC, which is known to interact directly with TRBV20-1^12^, and by TSST-1 (Fig. S3a, b). These results suggested that neither SpeC nor TSST-1 induced the downregulation and/or internalization of TRBV12-3/12-4^+^ TCRs. Similarly, the frequencies of TRBV12-3/12-4^+^ CD4^+^ and CD8^+^ T cells remained stable or even increased after stimulation with SEA, SEB, or SEC3 (Fig. 1b). None of the SAgs in our panel affected the lineage-defined frequencies of TRBV9^+^ cells, but consistent with a previous report^33^, stimulation with SEB or SEC3 led to a decrease in the frequencies of TRBV10-1/10-2/10-3^+^ CD4^+^ and CD8^+^ T cells (Fig. S3a, b).

Another SAg produced by *S. aureus*, namely SEE, is known to downregulate TRBV12-3/12-4^+^ TCRs^34,37^. In line with earlier observations^34^, we found that SEE-activated TRBV12-3/12-4^+^ CD4^+^ and CD8^+^ T cells at least as potently as SpeC and TSST-1 (Fig. 1c). However, these effects were associated with markedly decreased frequencies of TRBV12-3/12-4^+^ CD4^+^ and CD8^+^ T cells (Fig. 1d), potentially indicating a distinct mechanism of action^34,37^.

Collectively, these data revealed that TRBV12-3/12-4^+^ cells in the CD4^+^ and CD8^+^ lineages were activated by SpeC and TSST-1, despite the apparent failure of these SAgs to internalize the corresponding TCRs.

### SpeC and TSST-1 induce polyfunctional TRBV12-3/12-4^+^ CD4^+^ and CD8^+^ T cells

**Fig. 2:**
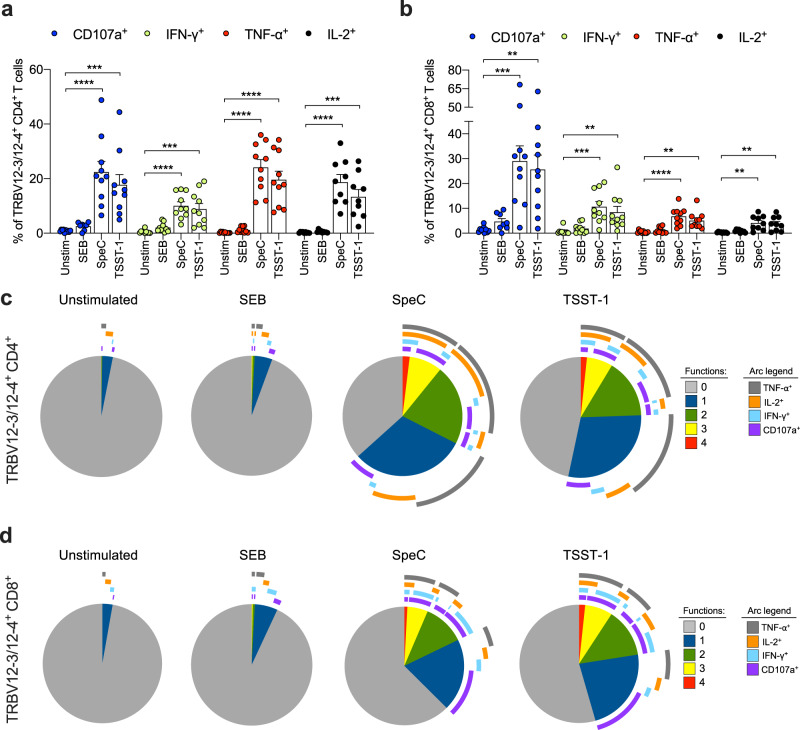
SpeC and TSST-1 induce polyfunctional TRBV12-3/12-4^+^ CD4^+^ and CD8^+^ T cells. **a**, **b** Frequency of CD107a^+^ (blue filled circles), IFN-γ^+^ (green filled circles), TNF-α^+^ (red filled circles), or IL-2^+^ cells (black filled circles) among TRBV12-3/12-4^+^ CD4^+^ (**a**) or CD8^+^ T cells (**b**) from human PBMCs cultured in medium alone (unstim) or stimulated for 24 h with SEB, SpeC, or TSST-1. Each dot represents one donor. Data are shown as mean ± SEM. **p < 0.01, ***p < 0.001, ****p < 0.0001. One-way ANOVA with Tukey’s post-hoc test. **c**, **d** Functional profiles of TRBV12-3/12-4^+^ CD4^+^ (**c**) or CD8^+^ T cells (**d**) from human PBMCs cultured in medium alone (unstimulated) or stimulated for 24 h with SEB, SpeC, or TSST-1. Pie chart segments from concatenated data (*n* = 4) represent the fractions of cells displaying the indicated numbers of functions (key). Arcs denote individual functions (key). The functional profiles elicited by SpeC and TSST-1 were significantly different from the corresponding functional profiles elicited by SEB (*p* < 0.05; permutation test).

SAgs are known to drive excessive cytokine production in a TRBV-specific manner^5,8,20,38–40^. To investigate this phenomenon in more detail, we measured the extent to which SpeC and TSST-1 induced the production of multiple cytokines (IFN-γ, TNF-α, and IL-2) and degranulation, quantified via the surface mobilization of CD107a^41^. In line with the activation data (Figs. 1a and S2c), we found that stimulation with SpeC or TSST-1 markedly increased the frequencies of TRBV12-3/12-4^+^ CD4^+^ and CD8^+^ T cells that mobilized CD107a or produced IFN-γ, TNF-α, or IL-2 (Fig. 2a, b). These effects were not observed after stimulation with SEB (Fig. 2a, b), which nonetheless elicited comparable responses among CD4^+^ and CD8^+^ T cells globally (Fig. S4a, b). Of note, higher frequencies of TNF-α^+^ or IL-2^+^ versus IFN-γ^+^ TRBV12-3/12-4^+^ CD4^+^ T cells were observed after stimulation with SpeC or TSST-1 (Fig. 2a), which predominantly elicited degranulation among TRBV12-3/12-4^+^ CD8^+^ T cells (Fig. 2b).

To extend these findings, we examined the dose and time dependency of activation and cytokine production in response to stimulation with SpeC or TSST-1. Each of these SAgs induced a dose-dependent increase in the frequencies of TRBV12-3/12-4^+^ CD4^+^ and CD8^+^ T cells that produced IFN-γ or TNF-α after 24 h (Fig. S5). Higher frequencies of IFN-γ^+^ TRBV12-3/12-4^+^ CD4^+^ and CD8^+^ T cells were observed after 48 h, but these temporal differences did not extend to the expression of CD69 or the production of TNF-α or IL-2 (Fig. S6a, b). Of note, similar frequencies of TRBV12-3/12-4^+^ CD4^+^ and CD8^+^ T cells produced IFN-γ or TNF-α after stimulation for 24 h with SEE, SpeC, or TSST-1, indicating largely equivalent functional potencies at a standardized concentration of 100 ng/mL (Fig. S6c, d).

Polyfunctional CD4^+^ and CD8^+^ T cells have been associated with enhanced immune protection^42^ but can also wane in the face of persistent antigen exposure^43^. We therefore concatenated the CD107a, IFN-γ, TNF-α, and IL-2 data (*n* = 4 donors) and examined the overall functional profiles of TRBV12-3/12-4^+^ CD4^+^ and CD8^+^ T cells using Boolean gating and Simplified Presentation of Incredibly Complex Evaluations (SPICE) software^44^. SpeC and TSST-1 both elicited monofunctional and polyfunctional TRBV12-3/12-4^+^ CD4^+^ and CD8^+^ T cell responses (Fig. 2c, d). Monofunctional responses were predominantly TNF-α^+^ among TRBV12-3/12-4^+^ CD4^+^ T cells and CD107a^+^ among TRBV12-3/12-4^+^ CD8^+^ T cells (Fig. 2c, d). In contrast, very few TRBV12-3/12-4^+^ CD4^+^ and CD8^+^ T cells were functionally responsive after stimulation with SEB (Fig. 2c, d).

Collectively, these results demonstrated that TRBV12-3/12-4^+^ cells in the CD4^+^ and CD8^+^ lineages were functionally responsive to SpeC and TSST-1, consistent with the synchronous upregulation of CD69.

### SpeC and TSST-1 upregulate coIRs among TRBV12-3/12-4^+^ CD4^+^ and CD8^+^ T cells

**Fig. 3:**
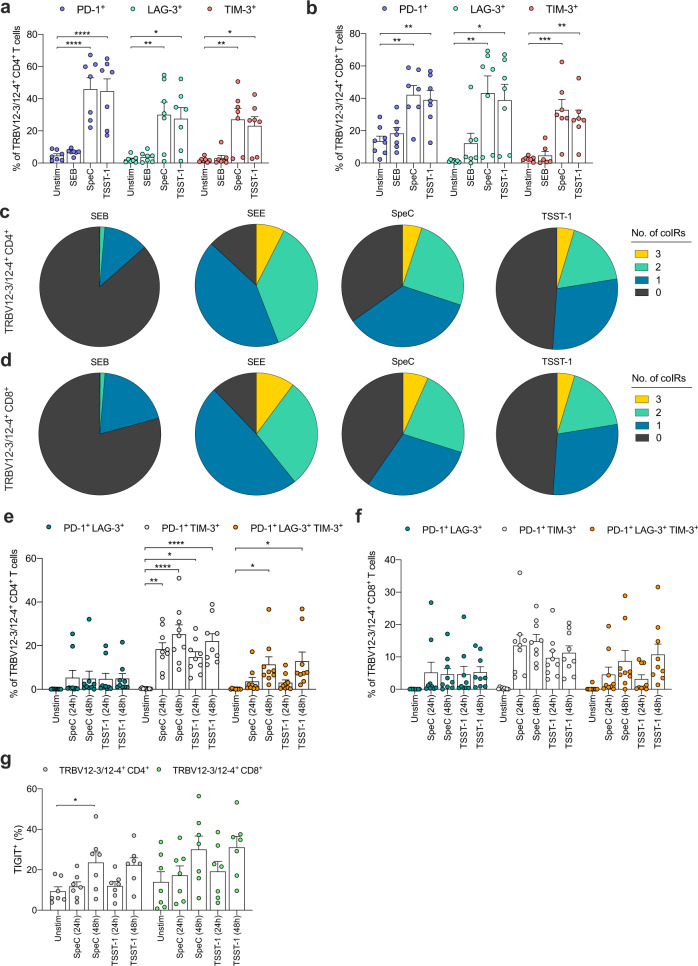
SpeC and TSST-1 upregulate coIRs among TRBV12-3/12-4^+^ CD4^+^ and CD8^+^ T cells. **a**, **b** Frequency of PD-1^+^ (mauve filled circles), LAG-3^+^ (aqua filled circles), or TIM-3^+^ cells (pink filled circles) among TRBV12-3/12-4^+^ CD4^+^ (**a**) or CD8^+^ T cells (**b**) from human PBMCs cultured in medium alone (unstim) or stimulated for 24 h with SEB, SpeC, or TSST-1. Each dot represents one donor. Data are shown as mean ± SEM. *p < 0.05, **p < 0.01, ***p < 0.001, ****p < 0.0001. One-way ANOVA with Tukey’s post-hoc test. **c**, **d** Expression profiles of coIRs (PD-1, LAG-3, and TIM-3) among TRBV12-3/12-4^+^ CD4^+^ (**c**) or CD8^+^ T cells (**d**) from human PBMCs stimulated for 24 h with SEB, SEE, SpeC, or TSST-1. Baseline expression was negligible. Pie chart segments from concatenated data (*n* = 6) represent the fractions of cells expressing the indicated numbers of coIRs (key). The expression profiles elicited by SEE, SpeC, and TSST-1 were significantly different from the corresponding expression profiles elicited by SEB (*p* < 0.05; permutation test). **e**, **f** Frequency of PD-1^+^ LAG-3^+^ (teal filled circles), PD-1^+^ TIM-3^+^ (gray filled circles), or PD-1^+^ LAG-3^+^ TIM-3^+^ cells (orange filled circles) among TRBV12-3/12-4^+^ CD4^+^ (**e**) or CD8^+^ T cells (**f**) from human PBMCs cultured in medium alone (unstim) or stimulated for 24 h or 48 h with SpeC or TSST-1. Each dot represents one donor. Data are shown as mean ± SEM. *p < 0.05, **p < 0.01, ****p < 0.0001. One-way ANOVA with Tukey’s post-hoc test. **g** Frequency of TIGIT^+^ cells among TRBV12-3/12-4^+^ CD4^+^ (gray filled circles) or CD8^+^ T cells (green filled circles) from human PBMCs cultured in medium alone (unstim) or stimulated for 24 h or 48 h with SpeC or TSST-1. Each dot represents one donor. Data are shown as mean ± SEM. *p < 0.05. One-way ANOVA with Tukey’s post-hoc test.

The upregulation of coIRs, such as programmed cell death protein-1 (PD-1), lymphocyte activation gene-3 (LAG-3), and T cell immunoglobulin and mucin domain-containing protein-3 (TIM-3), has been linked with T cell dysfunction and exhaustion, which can occur in the context of persistent antigen stimulation via the TCR^45–47^. Moreover, the functional capacity of MAIT cells can be perturbed by exposure to SEB, which concomitantly upregulates LAG-3 and TIM-3^8^. In line with these observations, we found that TRBV12-3/12-4^+^ CD4^+^ and CD8^+^ T cells more commonly expressed PD-1, LAG-3, or TIM-3 after stimulation with SpeC or TSST-1 (Fig. 3a, b). These effects were not observed after stimulation with SEB (Fig. 3a, b).

We then concatenated the PD-1, LAG-3, and TIM-3 data (*n* = 5 or 6 donors) and examined the overall coIR expression profiles of TRBV12-3/12-4^+^ CD4^+^ and CD8^+^ T cells as described above (Fig. 2c, d). The dataset was extended for these analyses to include SEE. Baseline expression of coIRs was negligible (Fig. S7a, b). In each lineage, a majority of TRBV12-3/12-4^+^ cells expressed at least one coIR after stimulation with SpeC or TSST-1, and ~5% of TRBV12-3/12-4^+^ cells expressed all three coIRs in response to each of these SAgs (Fig. 3c, d). Minimal effects were observed after stimulation with SEB, and slightly more potent effects were observed after stimulation with SEE (Fig. 3c, d).

The duration of exposure to SAgs can affect the expression patterns of multiple coIRs^8^. In our analyses, we found that most TRBV12-3/12-4^+^ CD4^+^ and CD8^+^ T cells that expressed two coIRs after stimulation for 24 h with SpeC or TSST-1 were either PD-1^+^ LAG-3^+^ or PD-1^+^ TIM-3^+^ (Fig. S7c, d). Higher frequencies of PD-1^+^ TIM-3^+^ or PD-1^+^ LAG-3^+^ TIM-3^+^ cells were observed after 48 h (Figs. 3e, f and S7a, b). At the same time point, we found that another coIR, namely T cell immunoreceptor with Ig and ITIM domains (TIGIT), was upregulated among TRBV12-3/12-4^+^ CD4^+^ and CD8^+^ T cells (Fig. 3g), which further exhibited a progressive loss of viability after stimulation with SpeC or TSST-1 (Fig. S7e).

Collectively, these data showed that multiple coIRs were upregulated among TRBV12-3/12-4^+^ cells in the CD4^+^ and CD8^+^ lineages after stimulation with SpeC or TSST-1, likely reflecting potent stimulation via the TCR.

### SpeC and TSST-1 stimulate TRBV12-3/12-4^+^ CD4^+^ and CD8^+^ memory T cells

**Fig. 4:**
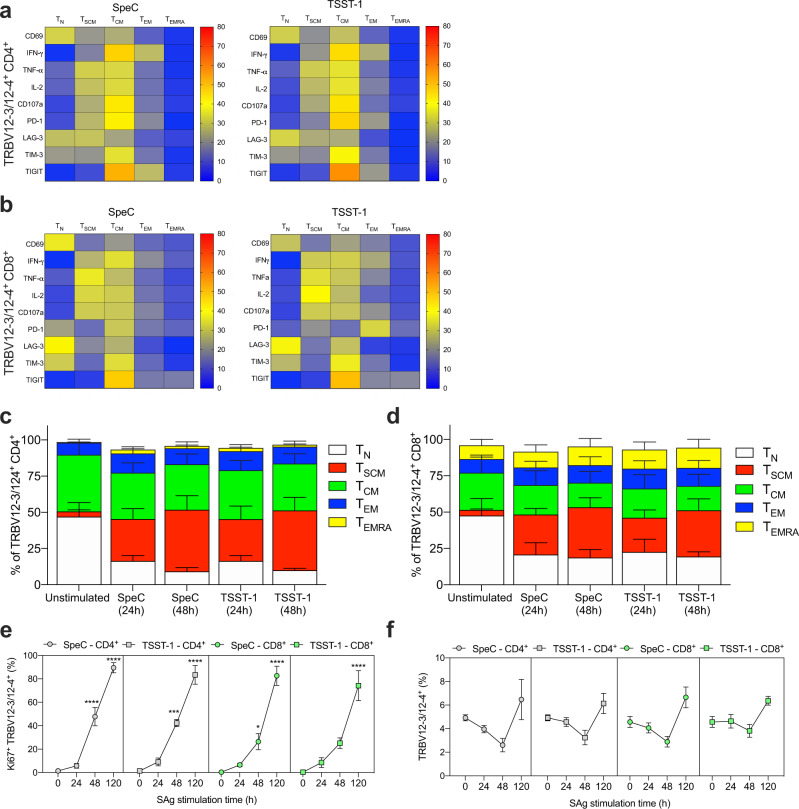
SpeC and TSST-1 stimulate TRBV12-3/12-4^+^ CD4^+^ and CD8^+^ memory T cells. **a**, **b** Heatmaps showing the frequency (key) of TRBV12-3/12-4^+^ CD4^+^ (**a**) or CD8^+^ T cells (**b**) expressing CD69, IFN-γ, TNF-α, IL-2, CD107a, PD-1, LAG-3, TIM-3, or TIGIT across the T_N_, T_SCM_, T_CM_, T_EM_, and T_EMRA_ subsets from human PBMCs stimulated for 24 h with SpeC or TSST-1. Concatenated data are shown (*n* = 4). **c**, **d** Stacked bar charts showing the frequency of TRBV12-3/12-4^+^ CD4^+^ (**c**) or CD8^+^ T cells (**d**) across the T_N_, T_SCM_, T_CM_, T_EM_, and T_EMRA_ subsets (key) from human PBMCs cultured in medium alone (unstimulated) or stimulated for 24 h or 48 h with SpeC or TSST-1 (*n* = 4). Data are shown as mean ± SEM. **e** Frequency of Ki67^+^ cells among TRBV12-3/12-4^+^ CD4^+^ (gray filled symbols) or CD8^+^ T cells (green filled symbols) from human PBMCs cultured in medium alone (0) or stimulated for 24 h, 48 h, or 120 h with SpeC (circles) or TSST-1 (squares) (*n* = 4). Data are shown as mean ± SEM. *p < 0.05, ***p < 0.001, ****p < 0.0001. One-way ANOVA with Tukey’s post-hoc test. **f** Frequency of TRBV12-3/12-4^+^ cells among CD4^+^ (gray filled symbols) or CD8^+^ T cells (green filled symbols) from human PBMCs cultured in medium alone (0) or stimulated for 24 h, 48 h, or 120 h with SpeC (circles) or TSST-1 (squares) (*n* = 4). Data are shown as mean ± SEM.

Immunological memory is thought to rely on long-lived and/or self-renewing subsets of stem-cell memory T (T_SCM_) and central memory T (T_CM_) cells^48–50^. Polyfunctionality is typically observed among the more differentiated T_CM_ and effector memory T (T_EM_) subsets^42,51,52^, but T_SCM_ cells are known to proliferate rapidly and often acquire similar functional attributes in response to SAgs^48,49^. To explore these phenotypic relationships in the context of SpeC and TSST-1, we quantified activation (CD69), coIR expression (PD-1, LAG-3, TIM-3, and TIGIT), and functionality (CD107a, IFN-γ, TNF-α, and IL-2) among discrete subsets of TRBV12-3/12-4^+^ CD4^+^ and CD8^+^ T cells. Established markers were used to distinguish naive T (T_N_) cells (CD45RA^+^ CD27^+^ CCR7^+^ CD95^−^), T_SCM_ cells (CD45RA^+^ CD27^+^ CCR7^+^ CD95^+^), T_CM_ cells (CD45RA^−^ CD27^+^ CCR7^+^ CD95^+^), T_EM_ cells (CD45RA^−^ CD27^−^ CCR7^−^ CD95^+^), and T_EM_ with revertant expression of CD45RA (T_EMRA_) cells (CD45RA^+^ CD27^−^ CCR7^−^ CD95^+^)^53,54^. We found that responsive TRBV12-3/12-4^+^ CD4^+^ and CD8^+^ T cells across all parameters were confined primarily to the T_SCM_ and T_CM_ compartments, although T_N_ cells not uncommonly expressed CD69, LAG-3, and TIM-3 after stimulation with SpeC or TSST-1 (Fig. 4a, b).

In line with these observations, we also found that TRBV12-3/12-4^+^ CD4^+^ and CD8^+^ T_SCM_ cells became more prevalent after stimulation with SpeC or TSST-1 (Fig. 4c, d). These expansions were accompanied by proportional declines in the corresponding T_N_ compartments after 24 h and 48 h (Fig. 4c, d) and globally increased expression frequencies of the proliferation marker Ki67 after 48 h and 120 h (Fig. 4e). Accordingly, the overall frequencies of TRBV12-3/12-4^+^ CD4^+^ and CD8^+^ T cells increased after 120 h (Fig. 4f), further confirming the specificity of SpeC and TSST-1.

Collectively, these results indicated that TRBV12-3/12-4^+^ T_SCM_ and T_CM_ cells in the CD4^+^ and CD8^+^ lineages were preferentially responsive to SpeC and TSST-1, leading to proliferation-induced distortions in subset composition alongside the apparently incongruous upregulation of multiple coIRs.

### SpeC and TSST-1 trigger TRBV12-3/12-4^+^ CD4^+^ and CD8^+^ T cells via HLA-DR

**Fig. 5:**
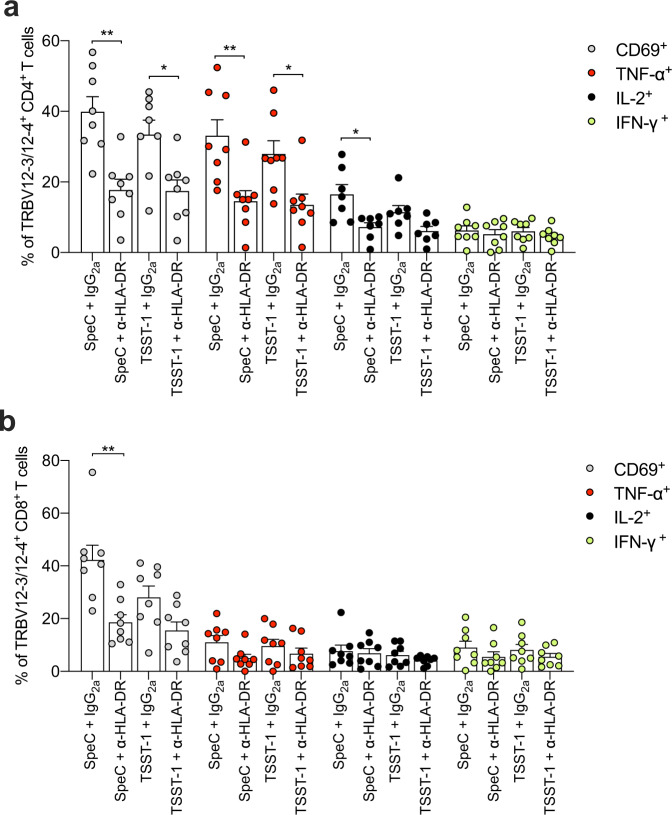
SpeC and TSST-1 trigger TRBV12-3/12-4^+^ CD4^+^ and CD8^+^ T cells via HLA-DR. **a**, **b** Frequency of CD69^+^ (gray filled circles), TNF-α^+^ (red filled circles), IL-2^+^ (black filled circles), or IFN-γ^+^ cells (green filled circles) among TRBV12-3/12-4^+^ CD4^+^ (**a**) or CD8^+^ T cells (**b**) from human PBMCs pretreated with anti-human HLA-DR or an isotype control antibody (IgG_2a_) and then stimulated for 24 h with SpeC or TSST-1. Each dot represents one donor. Data are shown as mean ± SEM. *p < 0.05, **p < 0.01. One-way ANOVA with Tukey’s post-hoc test.

Allotypic polymorphisms in HLA class II molecules, namely DQ and DR, can dramatically affect the functional consequences of defined interactions with SAgs^55^. SpeC is known to bind HLA-DR2a and HLA-DR4, and TSST-1 is known to bind HLA-DR1^56–58^. To investigate the role of these interactions in the context of SAg-induced activation via TRBV12-3/12-4^+^ TCRs, we used a previously characterized monoclonal antibody to block the functionality of HLA-DR^8^. We found that HLA-DR blockade inhibited the activation (CD69) and functional responsiveness (IFN-γ, TNF-α, and IL-2) of TRBV12-3/12-4^+^ CD4^+^ and CD8^+^ T cells relative to an isotype control antibody after stimulation with SpeC or TSST-1 (Fig. 5a, b). The corresponding effect sizes were broadly similar across conditions, lineages, and measured parameters, but significance was achieved more commonly among TRBV12-3/12-4^+^ CD4^+^ T cells, which responded more vigorously than TRBV12-3/12-4^+^ CD8^+^ T cells in the presence of functional HLA-DR (Fig. 5a, b).

Collectively, these data aligned with the known specificities of SpeC and TSST-1 for HLA class II molecules, confirming a requirement for HLA-DR functionality in the context of TRBV12-3/12-4^+^ TCRs.

### TSST-1 interacts with CD4^+^ and CD8^+^ T cells expressing TRBV12-3/12-4^+^ TCRs

SpeC and TSST-1 are known to engage TRBV20-1^+^ TCRs^10,12^. To explore the possibility that similar direct interactions occur in the context of TRBV12-3/12-4^+^ TCRs, we generated fluorescent tetrameric complexes of SEE, SpeC, and TSST-1. In flow cytometry experiments, we observed clear binding of the SEE tetramer but not the SpeC or TSST-1 tetramers to single TRBV12-3/12-4^+^ CD4^+^ and CD8^+^ T cells gated among PBMCs (Fig. S8a, b). Further gating on doublets to assess the impact of immune cell interactions^59^ revealed even clearer staining with the SEE tetramer and moderate staining with the TSST-1 tetramer but provided no evidence of a direct interaction between SpeC and TRBV12-3/12-4^+^ TCRs (Fig. S8a, c). This latter observation likely reflected a technical issue, because the SpeC tetramer also failed to bind TRBV20-1^+^ TCRs (Fig. S9a).

**Fig. 6:**
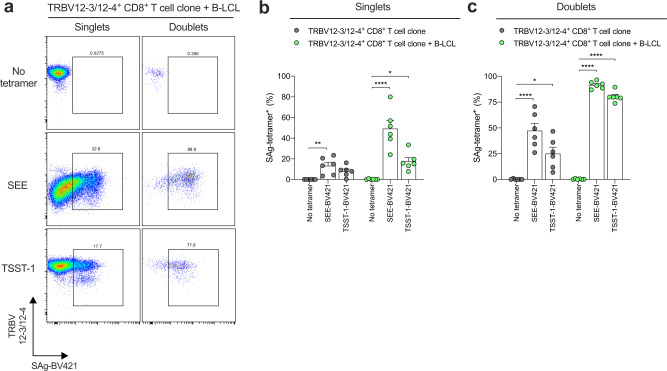
TSST-1 interacts with clonal CD8^+^ T cells expressing TRBV12-3/12-4^+^ TCRs. **a** Representative flow cytometry plots showing the frequency of SEE or TSST-1 tetramer^+^ events among clonal TRBV12-3/12-4^+^ CD8^+^ T cells cultured at a 1:1 ratio with the MR B-LCL. Plots are gated on live singlets (left) or doublets (right). **b**, **c** Frequency of SEE or TSST-1 tetramer^+^ events among clonal TRBV12-3/12-4^+^ CD8^+^ T cells gated as singlets (**b**) or doublets (**c**) in the absence (dark gray filled circles) or presence of the MR B-LCL (green filled circles). Each dot represents one experiment. Data are shown as mean ± SEM. *p < 0.05, **p < 0.01, ****p < 0.0001. One-way ANOVA with Tukey’s post-hoc test.

To corroborate these findings, we stained a TRBV12-3/12-4^+^ CD8^+^ T cell clone with the SEE and TSST-1 tetramers in the absence or presence of an HLA class II^+^ B-lymphoblastoid cell line (B-LCL) termed MR. Each tetramer exhibited a degree of binding to single TRBV12-3/12-4^+^ CD8^+^ T cells in the absence of MR, and the corresponding staining frequencies were enhanced dramatically in the presence of MR (Fig. 6a, b). Moreover, the TSST-1 tetramer engaged a TRBV20-1^+^ CD8^+^ T cell clone (Fig. S10a, b), consistent with earlier structural work^10^. Further gating on doublets revealed even more extensive staining of clonal TRBV12-3/12-4^+^ CD8^+^ T cells with the SEE and TSST-1 tetramers, especially in the presence of MR (Fig. 6a, c). No staining was observed with the SpeC tetramer (Fig. S9b), despite the functional potency of the corresponding recombinant protein, which triggered clonal TRBV12-3/12-4^+^ CD8^+^ T cells to produce IFN-γ at frequencies equivalent to those elicited by SEE (Fig. S9c).

Collectively, these data showed that TSST-1 interacted with CD4^+^ and CD8^+^ T cells expressing TRBV12-3/12-4^+^ TCRs, akin to SEE.

## Discussion

In this study, we used polychromatic flow cytometry to map TRBV-specific response patterns against a range of SAgs from *S. aureus* and *S. pyogenes*. Our data confirmed previous results and further showed that CD4^+^ and CD8^+^ T cells expressing TRBV12-3/12-4^+^ TCRs were highly responsive to SpeC and TSST-1, which elicited a full range of effector functions and induced the upregulation of multiple coIRs. Comparable biological responses were observed after exposure to SEE^34,37^. In addition, we found that TRBV12-3/12-4^+^ CD4^+^ and CD8^+^ T_SCM_ and T_CM_ cells were particularly susceptible to the stimulatory effects of SpeC and TSST-1, which required functional interactions with HLA-DR.

A number of staphylococcal SAgs, including SED, SEE, and various SE-like proteins, have been shown to exhibit specificity for TRBV12-3/12-4^+^ TCRs^34,60,61^. However, this particular interaction has not been reported previously for TSST-1, which stringently engages TRBV20-1^+^ TCRs^5,10,19,34,60^. Advances in flow cytometry and the availability of better reagents likely helped us identify this interaction. It is notable here that TSST-1 largely failed to induce the downregulation and/or internalization of TRBV12-3/12-4^+^ TCRs. This latter readout was used extensively in earlier studies to identify patterns of reactivity^33,34^. Other relatively insensitive techniques were also used previously, including various measurements of selective expansion/proliferation^19,60^. In contrast, our experimental approach relied primarily on the detection of functional responses, potentially facilitating the identification of biologically relevant interactions between SAgs and TRBV-defined TCRs.

An earlier study reported that TRBV12-3/12-4^+^ cells became activated in response to stimulation with SpeC^62^. However, this result was later questioned by the same group of investigators^63^, who suspected contamination of the original SAg preparation with SpeA, which is known to exhibit specificity for TRBV12-3/12-4^+^ TCRs^64,65^. In particular, they found that recombinant SpeC did not induce the secretion of IL-2 from Jurkat cells expressing TRBV12-3/12-4^+^ TCRs^63^, even in the presence of Raji cells to compensate for a lack of HLA-DQ and HLA-DR^66^. We found that TRBV12-3/12-4^+^ cells became fully activated after stimulation with recombinant SpeC, likely reflecting the provision of appropriate HLA class II molecules in the context of PBMCs. Of note, proliferation was delayed relative to activation, potentially explaining why this interaction was overlooked previously^36,67^. The mechanisms that underlie the stimulatory effects of SpeA, which can also target TRBV20-1^+^ TCRs^63,68^, and SpeC may nonetheless prove dichotomous for several reasons. In particular, the amino acid residues in SpeC that engage TRBV20-1^+^ TCRs are not conserved in SpeA^12^, and SpeC preferentially binds HLA-DR molecules rather than HLA-DQ molecules^69^, which are favored by SpeA^58^. Accordingly, SpeA and SpeC could use different mechanisms to engage TRBV12-3/12-4^+^ TCRs, potentially incorporating various HLA class II molecules in the corresponding ternary complexes, especially given our finding that SpeC-induced activation was not completely inhibited via antibody-mediated blockade of HLA-DR. It is also notable that we were unable to detect a physical interaction between tetrameric SpeC and cell surface-expressed TRBV12-3/12-4^+^ TCRs. This observation likely reflected a technical limitation, such as fluorochrome-associated steric hindrance, but could alternatively be explained by a very low monomeric interaction affinity, consistent with the finding that SpeC failed to induce the downregulation and/or internalization of TRBV12-3/12-4^+^ TCRs. Such a disconnect in terms of the affinity thresholds that govern activation and tetramer engagement has been described previously for conventional antigens^70–72^ and could similarly account for the lack of a visible interaction between tetrameric SpeC and cell surface-expressed TRBV20-1^+^ TCRs^12^.

TRBV-specific expansions associated with SAgs or SAg-like proteins have been described in the context of several life-threatening infectious conditions, including toxic shock syndrome^73^ and multisystem inflammatory syndrome in children, which has recently emerged as a severe complication of infection with SARS-CoV-2^74–76^. Highly focused expansions of TRBV12-3/12-4^+^ cells have also been described in Kawasaki disease^77^ and tuberculosis^78^. Of note, toxic shock syndrome can present as a complication of influenza virus infection or influenza-like respiratory illnesses^79^, and studies in mice have shown that SAgs can disrupt the influenza-specific memory T cell repertoire, negatively impacting subsequent immune protection^31,32^. This latter effect could have profound consequences in patients who develop bacterial superinfections^80^. Similarly, the targeted effects of SpeC and TSST-1 could anergize TRBV12-3/12-4-biased memory responses, potentially compromising immunity against CMV, dengue virus, and HIV^25,26,28,29^. Indeed, this scenario has been proposed to account for rapid disease progression in the context of infection with HIV, although no direct correlations were detected to support this notion^64,81^.

In summary, we have shown that SpeC and TSST-1 exhibit functional specificity for TRBV12-3/12-4^+^ TCRs, which are commonly expressed under physiological conditions and often mobilized to combat viral pathogens, such as CMV and HIV. Knowledge of these interactions could feasibly inform the etiology of several poorly understood infectious conditions and lead to the development of new molecular therapies for sepsis, which is one of the leading causes of death worldwide. On this basis, further studies are now warranted to dissect the atomic determinants of biological reactivity and, critically, to map the full extent to which SAgs engage the repertoire of human TCRs.

## Methods

### Cells

Venous blood samples were obtained from healthy volunteers, and buffy coats were purchased from the Welsh Blood Service. PBMCs were isolated via density gradient centrifugation using Histopaque-1077 (Sigma-Aldrich) and cryopreserved in fetal bovine serum containing 10% dimethyl sulfoxide (Sigma-Aldrich). TRBV-defined clones were generated from single flow-sorted CD8^+^ T cells via periodic stimulation with mixed irradiated allogeneic PBMCs, phytohemagglutinin (1 µg/mL), recombinant human IL-2 (200 IU/mL), and recombinant human IL-15 (25 ng/mL) in RPMI 1640 medium supplemented with 10% fetal bovine serum, 100 U/mL penicillin, 100 µg/mL streptomycin, and 2 mM l-glutamine (all from Thermo Fisher Scientific) (R10). The MR B-LCL was derived via in vitro transformation of primary B cells with EBV strain B95.8 and expressed HLA-DQ2, HLA-DQ8, and HLA-DR4. All cells were cultured under standard conditions in R10.

### Ethics

The use of venous blood samples from healthy volunteers was approved by the Cardiff University School of Medicine Research Ethics Committee (18/4). Written informed consent was obtained from all donors in accordance with the principles of the Declaration of Helsinki.

### Superantigen stimulation assays

PBMCs were thawed and rested for 24 h in R10. Cells were then seeded in 24-well culture plates (Thermo Fisher Scientific) at a density of >1 × 10^6^ cells/well and stimulated for 24 h (unless stated otherwise) with recombinant forms of SEA, SEB, SEC3, SEE, or TSST-1 from *S. aureus* or SpeC from *S. pyogenes* (Sigma-Aldrich or Toxin Technology), each at a dose of 100 ng/mL (unless stated otherwise) to align with previous work^8,33,40^. SAgs were pretested for purity at source via immunoassay and in-house via tandem mass spectrometry and SDS-PAGE. Unstimulated cells were used as negative controls. In mechanistic experiments, PBMCs were preincubated for 30 min with 10 μg/mL anti-human HLA-DR (clone L243; BioLegend) or 10 μg/mL mouse IgG_2a_ (clone MOPC-173; BioLegend). In functional experiments, anti-CD107a–BV785 (clone H4A3; BioLegend) was added to the cultures with each SAg^41^, and protein transport was blocked for the final 6 h with GolgiPlug (1:1,000; BD Biosciences) and GolgiStop (1:1,500; BD Biosciences).

### Superantigen tetramer binding assays

SAg tetramers were generated from biotinylated forms of SEE, SpeC, and TSST-1 (Toxin Technology) via conjugation at a 4:1 molar ratio to purified streptavidin labeled with BV421 (BioLegend). All tetramers were prepared immediately prior to experimentation. SAg tetramer stains were conducted using a standardized dose of 0.02 nmol per monomer for 30–60 min at 37 °C in R10.

### Flow cytometry

Cells were washed in Dulbecco’s phosphate-buffered saline (Thermo Fisher Scientific), labeled for 15–30 min at room temperature with Zombie Aqua (BioLegend), and blocked for 10 min at 4 °C with Human TruStain FcX (BioLegend). Surface stains were performed for 30 min at 4 °C using combinations of the following directly conjugated monoclonal antibodies: (i) anti-CCR7–FITC (clone 150503) from BD Biosciences; (ii) anti-CD27–PC5 (clone 1A4CD27) from Beckman Coulter; (iii) anti-CCR7–BV421 (clone G043H7), anti-CD3–APC/Fire 750 (clone SK7), anti-CD3–PE-Cy5 (clone SK7), anti-CD3–PerCP (clone SK7), anti-CD4–BV605 (clone OKT4), anti-CD8a–BV711 (clone RPA-T8), anti-CD25–APC/Fire 750 (clone BC96), anti-CD45RA–FITC (clone HI100), anti-CD45RA–PE/Dazzle 594 (clone HI100), anti-CD69–APC (clone FN50), anti-CD69–BV421 (clone FN50), anti-CD69–BV785 (clone FN50), anti-CD69–FITC (clone FN50), anti-CD69–PE (clone FN50), anti-CD95–PE (clone DX2), anti-LAG-3–FITC (clone 11C3C65), anti-LAG-3–PE-Cy7 (clone 11C3C65), anti-PD-1–BV605 (clone EH12.2H7), anti-PD-1–PE (clone EH12.2H7), anti-PD-1–PE/Dazzle 594 (clone EH12.2H7), anti-TIGIT–BV421 (clone A15153G), anti-TIGIT–BV605 (clone A15153G), anti-TIM-3–BV785 (clone F38-2E2), anti-TIM-3–PE (clone F38-2E2), anti-TIM-3–PE/Dazzle 594 (clone F38-2E2), anti-TCR Vβ8–APC (clone JR2), and anti-TCR Vβ8–PE-Cy7 (clone JR2) from BioLegend; (iv) anti-TCR Vβ1–APC-Vio770 (clone REA662), anti-TCR Vβ2–FITC (clone REA654), and anti-TCR Vβ2–PE-Vio770 (clone REA654) from Miltenyi Biotec; and (v) anti-CD4–PE-Cy5.5 (clone S3.5) and anti-TCR Vβ12–FITC (clone S511) from Thermo Fisher Scientific. Cytosolic/intranuclear expression of Ki67 was detected using anti-Ki67–FITC (clone B56; BD Biosciences) in conjunction with a Foxp3 Transcription Factor Staining Buffer Kit (Thermo Fisher Scientific). Intracellular cytokines were exposed using a Cytofix/Cytoperm Plus Fixation/Permeabilization Solution Kit (BD Biosciences) and stained for 30 min at 4 °C with combinations of the following directly conjugated monoclonal antibodies: (i) anti-IFN-γ–APC (clone B27), anti-IFN-γ–FITC (clone 4S.B3), anti-IL-2–PE/Dazzle 594 (clone MQ1-17H12), anti-TNF-α–BV605 (clone MAb11), and anti-TNF-α–BV785 (clone MAb11) from BioLegend; and (ii) anti-TNF-α–APC-Vio770 (clone cA2) from Miltenyi Biotec. All flow cytometry panels were validated using individually stained Anti-Mouse Ig, κ/Negative Control Particles (BD Biosciences). Data were acquired using a modified FACS Aria II (BD Biosciences) or an Attune NxT (Thermo Fisher Scientific) and analyzed using FlowJo software version 9.9.6 or version 10.7.1 (FlowJo LLC).

### Statistics and reproducibility

Differences among groups were evaluated using a one-way ANOVA with Tukey’s post-hoc test in Prism version 8.4.3 (GraphPad). Significance was assigned at *p* < 0.05. Functional profiles and concatenated phenotypic datasets were compared using the permutation test over 10,000 iterations in SPICE software version 6.0^44^. Reproducibility was assessed using multiple independent samples of PBMCs.

### Reporting summary

Further information on research design is available in the Nature Portfolio Reporting Summary linked to this article.

## Supplementary information

Supplemental Figures

Reporting Summary

## Data Availability

Source data will be made available on request from the corresponding author, James E. McLaren.
