# Supplementary material for: The superantigens SpeC and TSST-1 specifically activate TRBV12-3/12-4+ memory T cells
Source: Commun Biol. 2023 Jan 20;6:78. doi: 10.1038/s42003-023-04420-1 (PMC9854414; doi:10.1038/s42003-023-04420-1)
Supplement: Supplementary file 1 — Supplemental Figures [file 42003_2023_4420_MOESM1_ESM.pdf]

SUPPLEMENTARY INFORMATION

The superantigens SpeC and TSST-1 specifically activate TRBV12-3/12-4$^+$ memory T cells

Freya R. Shepherd[1], Kate Davies[1], Kelly L. Miners[1], Sian Llewellyn-Lacey[1], Simon Kollnberger[1], James E. Redman[2], Melissa M. Grant[3], Kristin Ladell[1], David A. Price[1,4], and James E. McLaren[1,*]

[1]Division of Infection and Immunity, School of Medicine, Cardiff University, Cardiff, UK

[2]School of Chemistry, Cardiff University, Cardiff, UK

[3]School of Dentistry, Institute of Clinical Sciences, University of Birmingham, Birmingham, UK

[4]Systems Immunity Research Institute, School of Medicine, Cardiff University, Cardiff, UK

*Corresponding author: mclarenje@cardiff.ac.uk (J.E.M.)

Keywords: CD4$^+$ T cells, CD8$^+$ T cells, SpeC, superantigen, TCR, TRBV12-3/12-4, TSST-1.

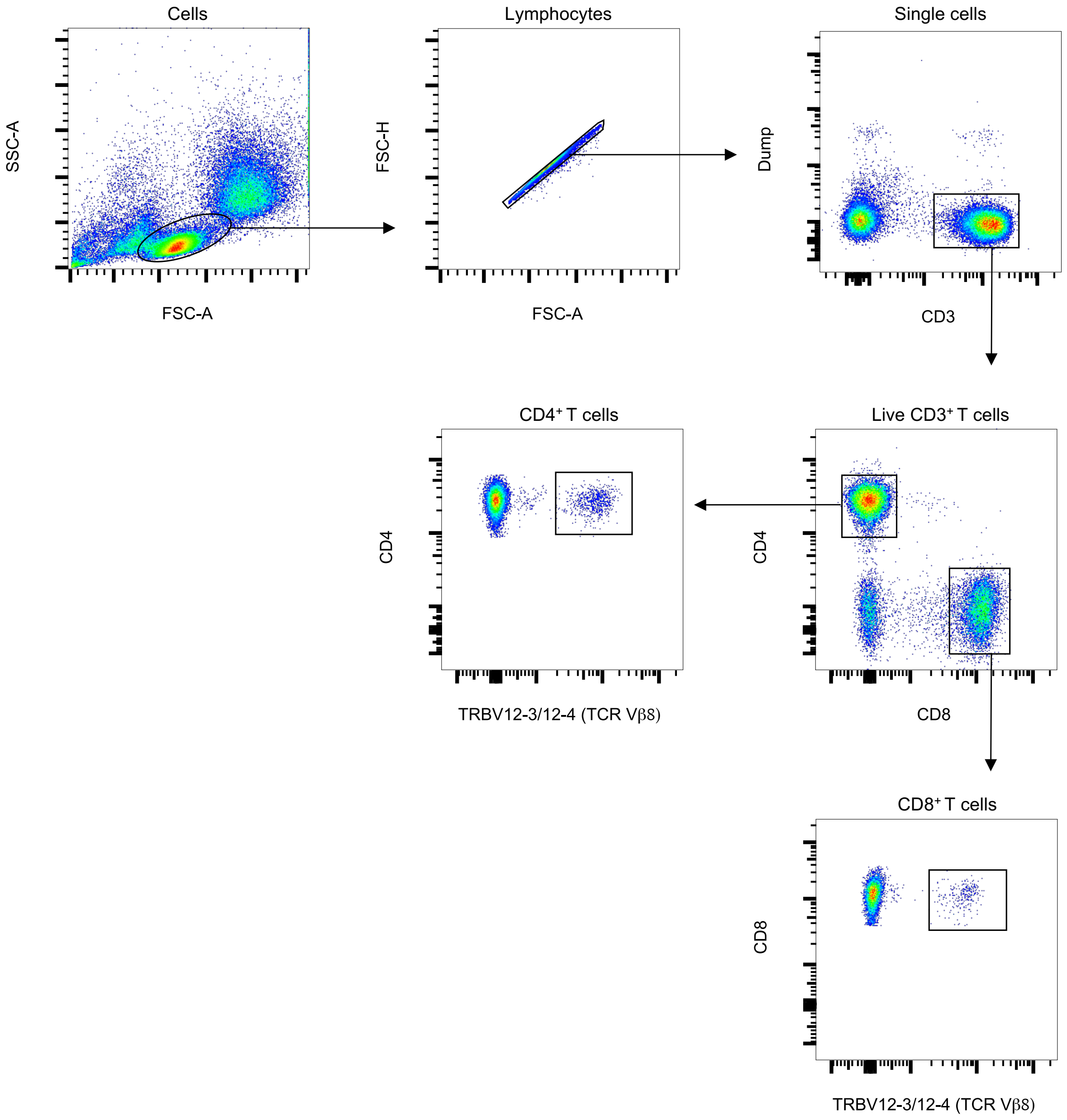

Figure S1 – Flow cytometric gating strategy for the identification of CD4$^+$ and CD8$^+$ T cells expressing TRBV12-3/12-4$^+$ TCRs

Bivariate flow cytometry plots showing the serial identification of lymphocytes, single cells, viable CD3$^+$ cells, and CD4$^+$ and CD8$^+$ T cells expressing TRBV12-3/12-4$^+$ TCRs.

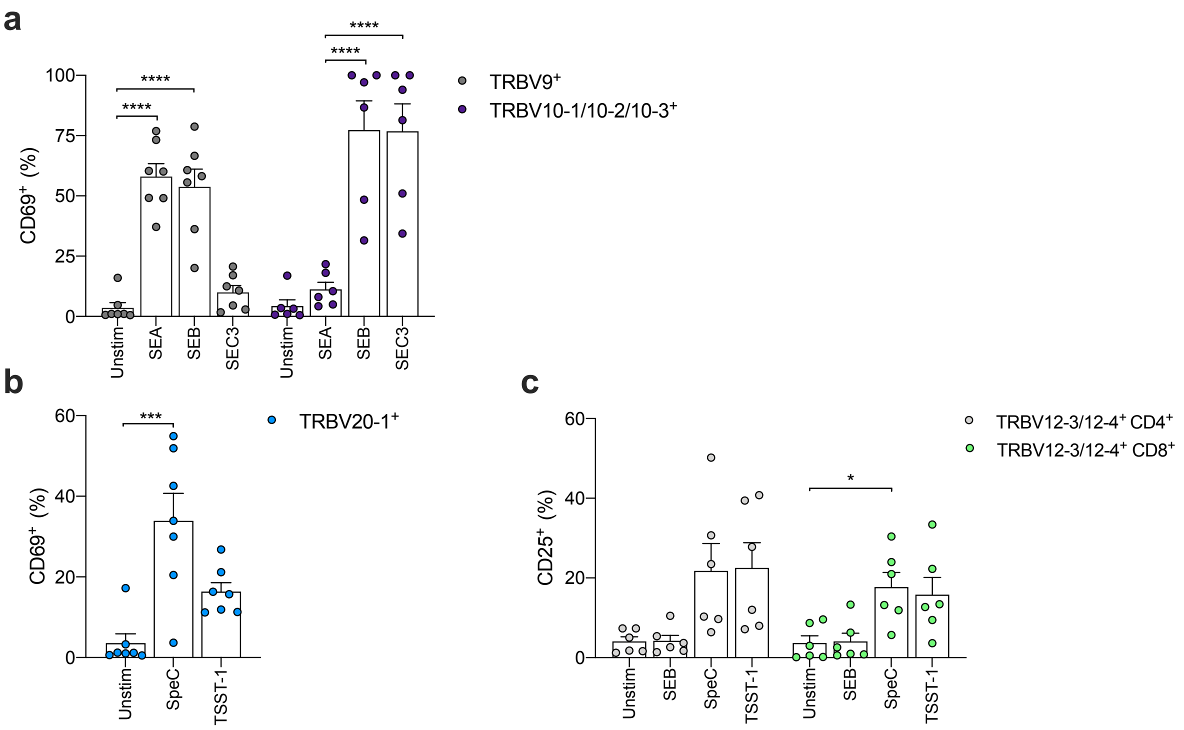

Figure S2 – TRBV-specific CD4$^+$ and CD8$^+$ T cell activation in response to bacterial SAgs

(**a**) Frequency of CD69$^+$ cells among TRBV9$^+$ (dark grey filled circles) or TRBV10-1/10-2/10-3$^+$ CD4$^+$ T cells (purple filled circles) from human PBMCs cultured in medium alone (unstim) or stimulated for 24 h with SEA, SEB, or SEC3. Each dot represents one donor. Data are shown as mean ± SEM. ****$p < 0.0001$. One-way ANOVA with Tukey's post-hoc test. (**b**) Frequency of CD69$^+$ cells among TRBV20-1$^+$ CD4$^+$ T cells (blue filled circles) from human PBMCs cultured in medium alone (unstim) or stimulated for 24 h with SpeC or TSST-1. Each dot represents one donor. Data are shown as mean ± SEM. ***$p < 0.001$. One-way ANOVA with Tukey's post-hoc test. (**c**) Frequency of CD25$^+$ cells among TRBV12-3/12-4$^+$ CD4$^+$ (grey filled circles) or CD8$^+$ T cells (green filled circles) from human PBMCs cultured in medium alone (unstim) or stimulated for 24 h with SEB, SpeC, or TSST-1. Each dot represents one donor. Data are shown as mean ± SEM. *$p < 0.05$. One-way ANOVA with Tukey's post-hoc test.

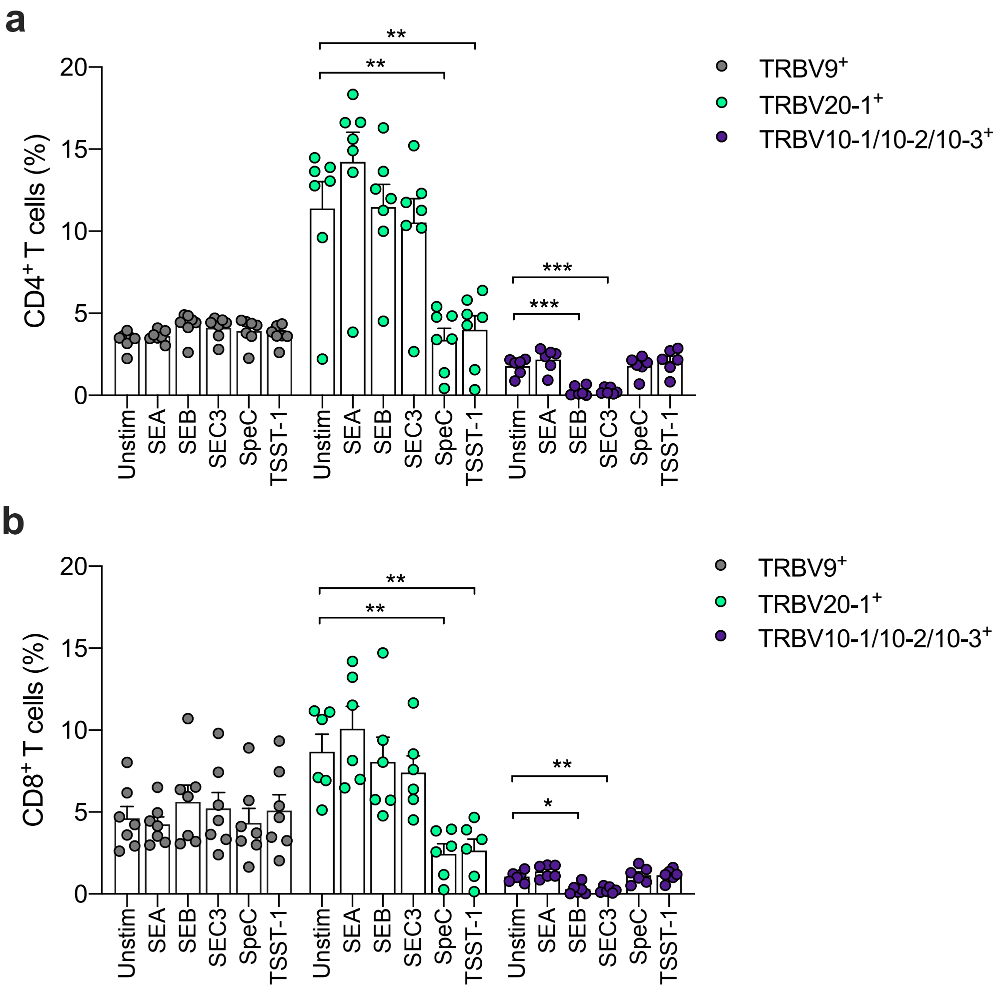

Figure S3 – SAg-driven downregulation of TRBV-defined TCRs

(**a**, **b**) Frequency of $TRBV9^+$ (dark grey filled circles), TRBV20-1^+ (green filled circles), or TRBV10-1/10-2/10-3^+ cells (purple filled circles) among $CD4^+$ (a) or $CD8^+$ T cells (b) from human PBMCs cultured in medium alone (unstim) or stimulated for 24 h with SEA, SEB, SEC3, SpeC, or TSST-1. Each dot represents one donor. Data are shown as mean ± SEM. *$p < 0.05$, **$p < 0.01$, ***$p < 0.001$. One-way ANOVA with Tukey's post-hoc test.

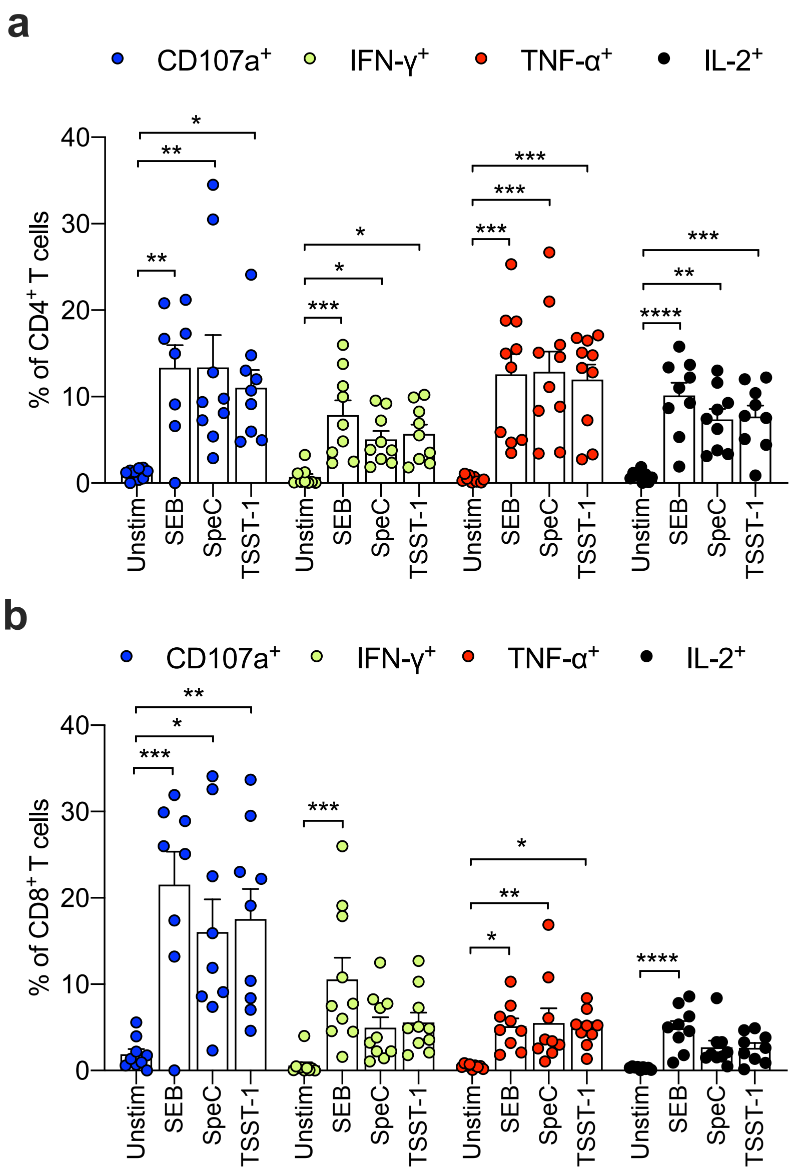

Figure S4 – SAg-driven cytokine production among CD4$^+$ and CD8$^+$ T cells

(**a**, **b**) Frequency of CD107a$^+$ (blue filled circles), IFN-γ$^+$ (green filled circles), TNF-α$^+$ (red filled circles), or IL-2$^+$ cells (black filled circles) among CD4$^+$ (a) or CD8$^+$ T cells (b) from human PBMCs cultured in medium alone (unstim) or stimulated for 24 h with SEB, SpeC, or TSST-1. Each dot represents one donor. Data are shown as mean ± SEM. *$p < 0.05$, **$p < 0.01$, ***$p < 0.001$, ****$p < 0.0001$. One-way ANOVA with Tukey's post-hoc test.

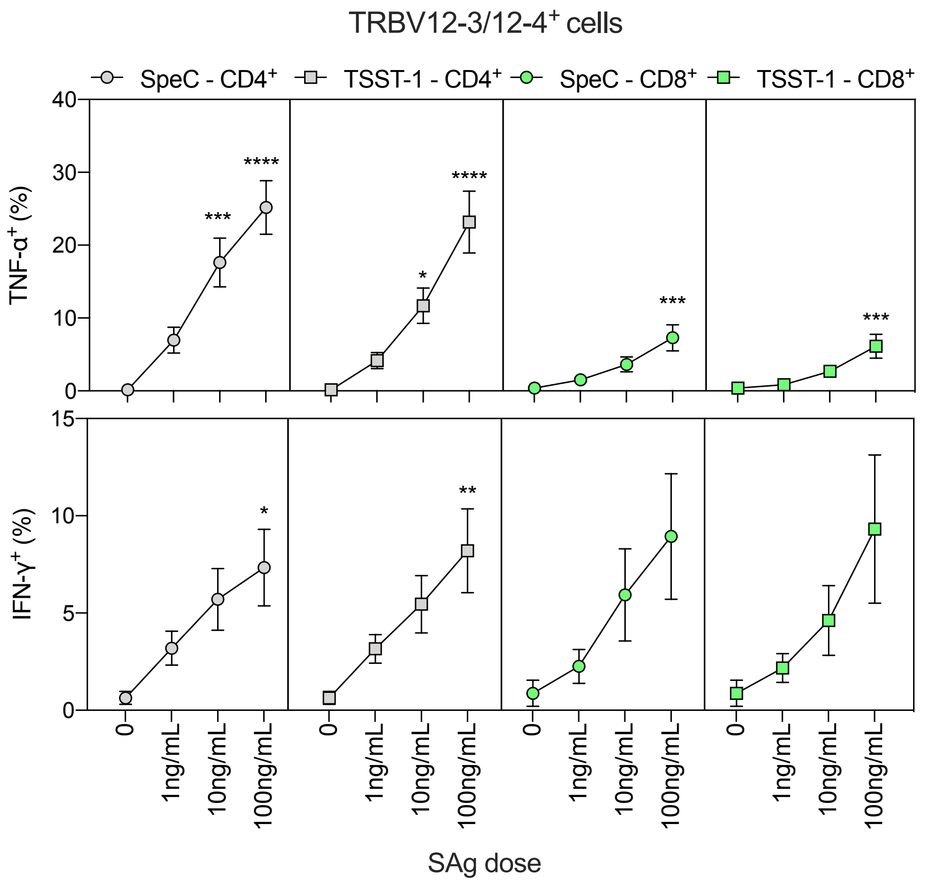

Figure S5 – Dose dependency of cytokine production among TRBV12-3/12-4$^+$ CD4$^+$ and CD8$^+$ T cells stimulated with SpeC or TSST-1

Frequency of TNF-α$^+$ (top) or IFN-γ$^+$ cells (bottom) among TRBV12-3/12-4$^+$ CD4$^+$ (grey filled symbols) or CD8$^+$ T cells (green filled symbols) from human PBMCs cultured in medium alone (0) or stimulated for 24 h with SpeC (circles) or TSST-1 (squares) at concentrations of 1 ng/mL, 10 ng/mL, or 100 ng/mL ($n = 6$). Data are shown as mean ± SEM. *$p < 0.05$, **$p < 0.01$, ***$p < 0.001$, ****$p < 0.0001$. One-way ANOVA with Tukey's post-hoc test.

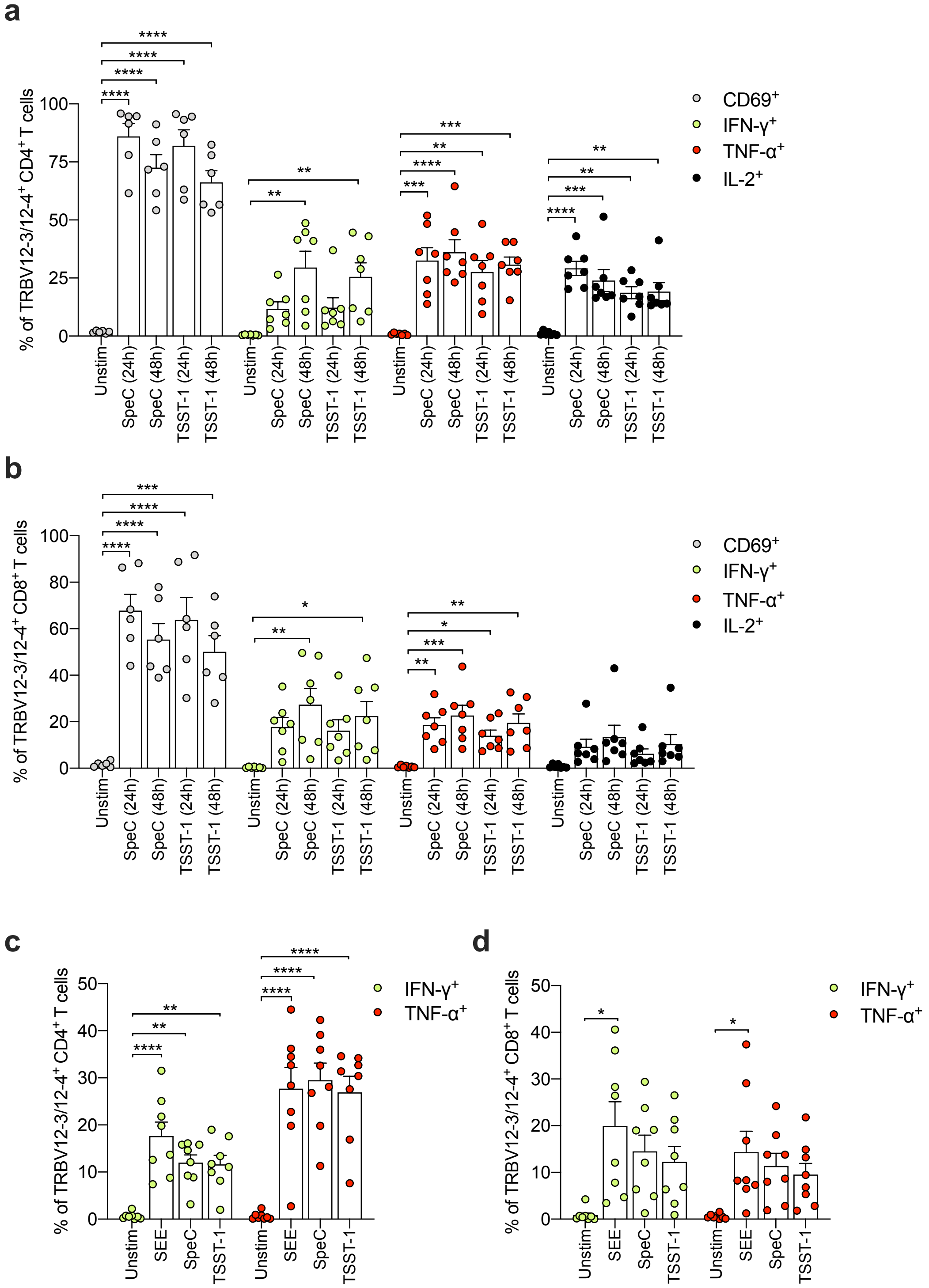

Figure S6 – Functional profiles of TRBV12-3/12-4$^+$ CD4$^+$ and CD8$^+$ T cells stimulated with SEE, SpeC, or TSST-1

(**a**, **b**) Frequency of CD69$^+$ (grey filled circles), IFN-γ$^+$ (green filled circles), TNF-α$^+$ (red filled circles), or IL-2$^+$ cells (black filled circles) among TRBV12-3/12-4$^+$ CD4$^+$ (a) or CD8$^+$ T cells

(b) from human PBMCs cultured in medium alone (unstim) or stimulated for 24 h or 48 h with SpeC or TSST-1. Each dot represents one donor. Data are shown as mean ± SEM. *p < 0.05, **p < 0.01, ***p < 0.001, ****p < 0.0001. One-way ANOVA with Tukey's post-hoc test. (**c**, **d**) Frequency of IFN-γ^+ (green filled circles) or TNF-α^+ cells (red filled circles) among TRBV12-3/12-4$^+$ CD4$^+$ (c) or CD8$^+$ T cells (d) from human PBMCs cultured in medium alone (unstim) or stimulated for 24 h with SEE, SpeC, or TSST-1. Each dot represents one donor. Data are shown as mean ± SEM. *p < 0.05, **p < 0.01, ****p < 0.0001. One-way ANOVA with Tukey's post-hoc test.

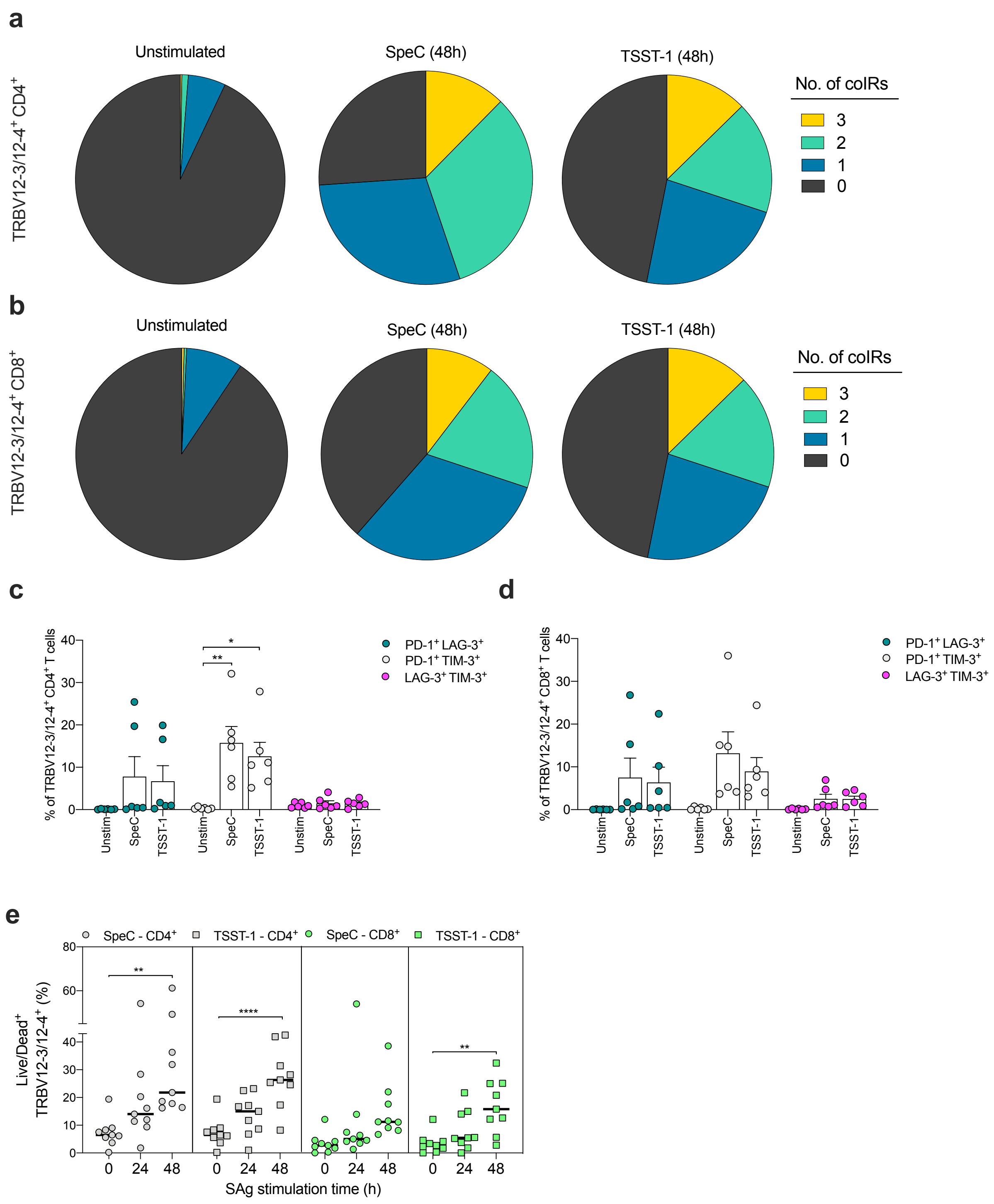

Figure S7 – Upregulation of coIRs among TRBV12-3/12-4$^+$ CD4$^+$ and CD8$^+$ T cells stimulated with SpeC or TSST-1

(**a**, **b**) Expression profiles of coIRs (PD-1, LAG-3, and TIM-3) among TRBV12-3/12-4$^+$ CD4$^+$ (a) or CD8$^+$ T cells (b) from human PBMCs cultured in medium alone (unstim) or stimulated

for 48 h with SpeC or TSST-1. Pie chart segments from concatenated data (n = 6) represent the fractions of cells expressing the indicated numbers of coIRs (key). (**c**, **d**) Frequency of PD-1^+ LAG-3^+ (teal filled circles), PD-1^+ TIM-3^+ (grey filled circles), or LAG-3^+ TIM-3^+ cells (pink filled circles) among TRBV12-3/12-4^+ $CD4^+$ (c) or $CD8^+$ T cells (d) from human PBMCs cultured in medium alone (unstim) or stimulated for 24 h with SpeC or TSST-1. Each dot represents one donor. Data are shown as mean ± SEM. *p < 0.05, **p < 0.01. One-way ANOVA with Tukey's post-hoc test. (**e**) Frequency of Live/Dead Zombie $Aqua^+$ cells among TRBV12-3/12-4^+ $CD4^+$ (grey filled symbols) or $CD8^+$ T cells (green filled symbols) from human PBMCs cultured in medium alone (0) or stimulated for 24 h or 48 h with SpeC (circles) or TSST-1 (squares). Each dot represents one donor. Data are shown as mean ± SEM. **p < 0.01, ****p < 0.0001. One-way ANOVA with Tukey's post-hoc test.

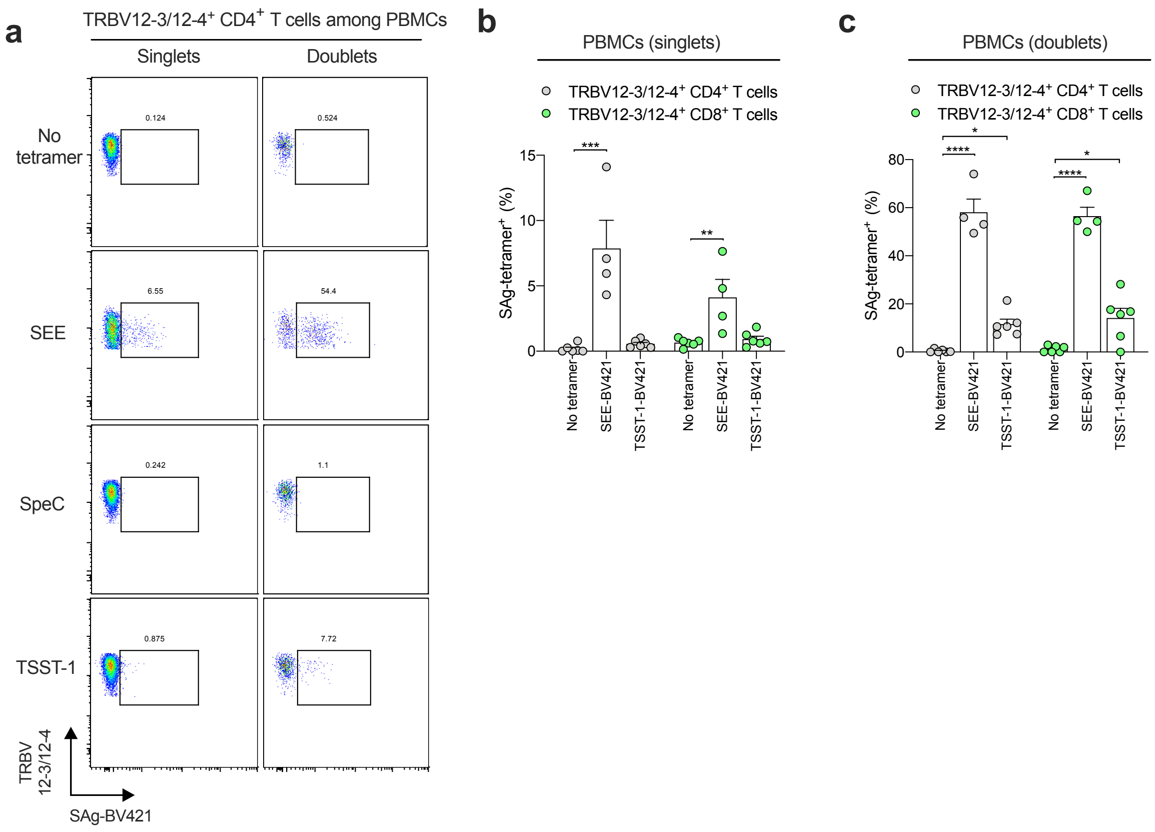

Figure S8 – TSST-1 interacts with CD4^{+} and CD8^{+} T cells expressing TRBV12-3/12-4^{+} TCRs

(**a**) Representative flow cytometry plots showing the frequency of SEE, SpeC, or TSST-1 tetramer^{+} events among TRBV12-3/12-4^{+} CD4^{+} T cells from human PBMCs. Plots are gated on live singlets (left) or doublets (right). (**b**, **c**) Frequency of SEE or TSST-1 tetramer^{+} events among TRBV12-3/12-4^{+} CD4^{+} (grey filled circles) or CD8^{+} T cells (green filled circles) gated as singlets (b) or doublets (c) from human PBMCs. Each dot represents one donor. Data are shown as mean ± SEM. *p < 0.05, **p < 0.01, ***p < 0.001, ****p < 0.0001. One-way ANOVA with Tukey's post-hoc test.

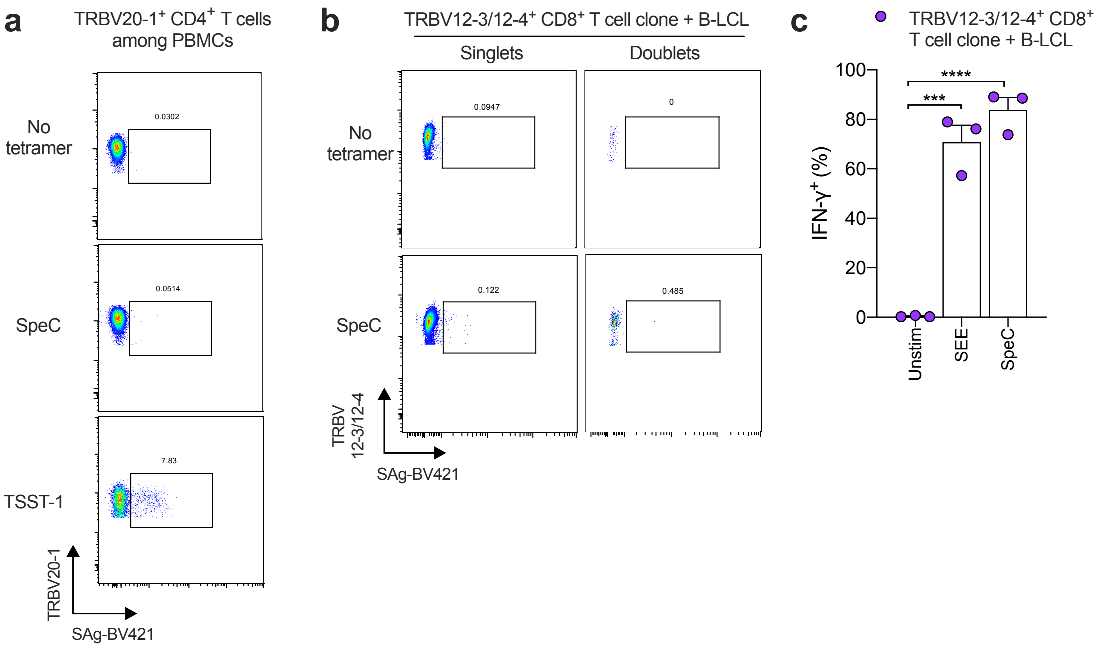

Figure S9 – SpeC elicits functional responses in the absence of an observable interaction with TRBV12-3/12-4$^+$ TCRs

(**a**) Representative flow cytometry plots showing the frequency of SpeC or TSST-1 tetramer$^+$ events among single TRBV20-1$^+$ CD4$^+$ T cells from human PBMCs. (**b**) Representative flow cytometry plots showing the frequency of SpeC tetramer$^+$ events among clonal TRBV12-3/12-4$^+$ CD8$^+$ T cells cultured at a 1:1 ratio with the MR B-LCL. Plots are gated on live singlets (left) or doublets (right). (**c**) Frequency of IFN-γ$^+$ cells (purple filled circles) among clonal TRBV12-3/12-4$^+$ CD8$^+$ T cells cultured at a 1:1 ratio with the MR B-LCL in medium alone (unstim) or stimulated for 24 h with SEE or SpeC. Each dot represents one experiment. Data are shown as mean ± SEM. ***$p < 0.001$, ****$p < 0.0001$. One-way ANOVA with Tukey's post-hoc test.

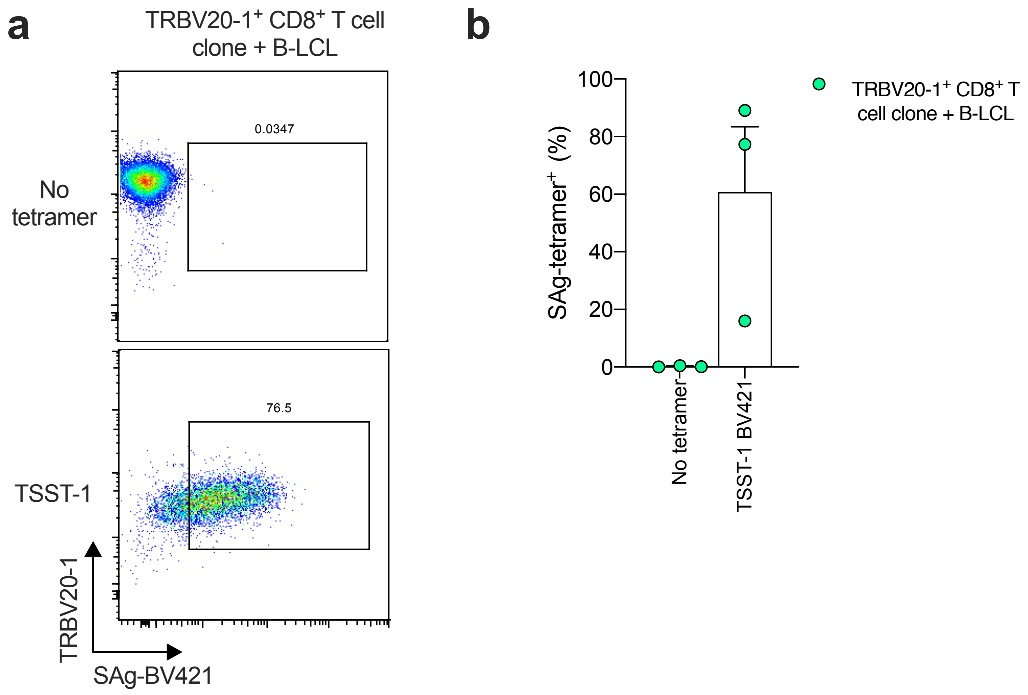

Figure S10 – TSST-1 interacts with clonal CD8⁺ T cells expressing TRBV20-1⁺ TCRs

(**a**) Representative flow cytometry plots showing the frequency of TSST-1 tetramer$^+$ events among single clonal TRBV20-1$^+$ CD8$^+$ T cells cultured at a 1:1 ratio with the MR B-LCL. (**b**) Frequency of TSST-1 tetramer$^+$ events (green filled circles) among single clonal TRBV20-1$^+$ CD8$^+$ T cells cultured at a 1:1 ratio with the MR B-LCL. Each dot represents one experiment. Data are shown as mean ± SEM.
