# Supplementary material for: The superantigens SpeC and TSST-1 specifically activate TRBV12-3/12-4+ memory T cells
Source: Commun Biol. 2023 Jan 20;6:78. doi: 10.1038/s42003-023-04420-1 (PMC9854414; doi:10.1038/s42003-023-04420-1)
Supplement: Supplementary file 2 — Reporting Summary [file 42003_2023_4420_MOESM2_ESM.pdf]

nature portfolio

Corresponding author(s): DR. JAMES MCLAREN

Last updated by author(s): Dec 20, 2022

Reporting Summary

Nature Portfolio wishes to improve the reproducibility of the work that we publish. This form provides structure for consistency and transparency in reporting. For further information on Nature Portfolio policies, see our Editorial Policies and the Editorial Policy Checklist.

Statistics

For all statistical analyses, confirm that the following items are present in the figure legend, table legend, main text, or Methods section.

n/a	Confirmed	
☐	☒	The exact sample size (*n*) for each experimental group/condition, given as a discrete number and unit of measurement
☐	☒	A statement on whether measurements were taken from distinct samples or whether the same sample was measured repeatedly
☐	☒	The statistical test(s) used AND whether they are one- or two-sided<br>*Only common tests should be described solely by name; describe more complex techniques in the Methods section.*
☒	☐	A description of all covariates tested
☐	☒	A description of any assumptions or corrections, such as tests of normality and adjustment for multiple comparisons
☐	☒	A full description of the statistical parameters including central tendency (e.g. means) or other basic estimates (e.g. regression coefficient) AND variation (e.g. standard deviation) or associated estimates of uncertainty (e.g. confidence intervals)
☒	☐	For null hypothesis testing, the test statistic (e.g. *F*, *t*, *r*) with confidence intervals, effect sizes, degrees of freedom and *P* value noted<br>*Give P values as exact values whenever suitable.*
☒	☐	For Bayesian analysis, information on the choice of priors and Markov chain Monte Carlo settings
☒	☐	For hierarchical and complex designs, identification of the appropriate level for tests and full reporting of outcomes
☒	☐	Estimates of effect sizes (e.g. Cohen's *d*, Pearson's *r*), indicating how they were calculated

Our web collection on statistics for biologists contains articles on many of the points above.

Software and code

Policy information about availability of computer code

Data collection	BD FACSDiva (v8.0) and ThermoFisher Attune NxT software was used to acquire flow cytometry data.
Data analysis	Software used in this study included: FlowJo (v9.9.6 or 10.7.1; TreeStar); GraphPad Prism (v8.4.3).

For manuscripts utilizing custom algorithms or software that are central to the research but not yet described in published literature, software must be made available to editors and reviewers. We strongly encourage code deposition in a community repository (e.g. GitHub). See the Nature Portfolio guidelines for submitting code & software for further information.

Data

Policy information about availability of data

All manuscripts must include a data availability statement. This statement should provide the following information, where applicable:

- Accession codes, unique identifiers, or web links for publicly available datasets
- A description of any restrictions on data availability
- For clinical datasets or third party data, please ensure that the statement adheres to our policy

The datasets generated during the current study are available from the corresponding author on reasonable request.

Field-specific reporting

Please select the one below that is the best fit for your research. If you are not sure, read the appropriate sections before making your selection.

☒ Life sciences ☐ Behavioural & social sciences ☐ Ecological, evolutionary & environmental sciences

For a reference copy of the document with all sections, see nature.com/documents/nr-reporting-summary-flat.pdf

Life sciences study design

All studies must disclose on these points even when the disclosure is negative.

Sample size	Sample sizes were not pre-determined by calculation and were based on the availability of samples.
Data exclusions	No data were excluded
Replication	Reproducibility was performed repeating experiments in individual donors
Randomization	Randomization was not relevant to this study
Blinding	Blinding was not relevant to this study

Reporting for specific materials, systems and methods

We require information from authors about some types of materials, experimental systems and methods used in many studies. Here, indicate whether each material, system or method listed is relevant to your study. If you are not sure if a list item applies to your research, read the appropriate section before selecting a response.

Materials & experimental systems

n/a	Involved in the study	
☐	☒	Antibodies
☒	☐	Eukaryotic cell lines
☒	☐	Palaeontology and archaeology
☒	☐	Animals and other organisms
☒	☐	Human research participants
☒	☐	Clinical data
☒	☐	Dual use research of concern

Methods

n/a	Involved in the study	
☒	☐	ChIP-seq
☐	☒	Flow cytometry
☒	☐	MRI-based neuroimaging

Antibodies

Antibodies used	(i) anti-CCR7–FITC (clone 150503) and anti-Ki67–FITC (clone B56) from BD Biosciences; (ii) anti-CD27–PC5 (clone 1A4CD27) from Beckman Coulter; (iii) anti-CCR7–BV421 (clone G043H7), anti-CD3–APC/Fire 750 (clone SK7), anti-CD3–PE-Cy5 (clone SK7), anti-CD3–PerCP (clone SK7), anti-CD4–BV605 (clone OKT4), anti-CD8a–BV711 (clone RPA-T8), anti-CD25–APC/Fire 750 (clone BC96), anti-CD45RA–FITC (clone HI100), anti-CD45RA–PE/Dazzle 594 (clone HI100), anti-CD69–APC (clone FN50), anti-CD69–BV421 (clone FN50), anti-CD69–BV785 (clone FN50), anti-CD69–FITC (clone FN50), anti-CD69–PE (clone FN50), anti-CD95–PE (clone DX2), anti-IFN-g–APC (clone B27), anti-IFN-g–FITC (clone 4S.B3), anti-IL-2–PE/Dazzle 594 (clone MQ1-483 17H12), anti-LAG-3–FITC (clone 11C3C65), anti-LAG-3–PE-Cy7 (clone 11C3C65), anti-PD-1–BV605 (clone EH12.2H7), anti-PD-1–PE (clone EH12.2H7), anti-PD-1–PE/Dazzle 594 (clone EH12.2H7), anti-TIGIT–BV421 (clone A15153G), anti-TIGIT–BV605 (clone A15153G), anti-TIM-3–BV785 (clone F38-2E2), anti-TIM-3–PE (clone F38-2E2), anti-TIM-3–PE/Dazzle 594 (clone F38-2E2), anti-TCR Vβ8–APC (clone JR2), and anti-TCR Vβ8–PE-Cy7 (clone JR2), anti-TNF-a–BV605 (clone MAb11), anti-TNF-a–BV785 (clone MAb11)from BioLegend; (iv) anti-TCR Vβ1–APC-Vio770 (clone REA662), anti-TCR Vβ2–FITC (clone REA654), and anti-TCR Vβ2–PE-Vio770 (clone REA654), anti-TNF-a–APC-Vio770 (clone cA2) from Miltenyi Biotec; and (v) anti-CD4–PE-Cy5.5 (clone S3.5), anti-TCR Vβ12–FITC (clone S511) from Thermo Fisher Scientific.
Validation	Antibodies were commercially obtained and not validated independently

Flow Cytometry

Plots

Confirm that:

☒ The axis labels state the marker and fluorochrome used (e.g. CD4-FITC).

☒ The axis scales are clearly visible. Include numbers along axes only for bottom left plot of group (a 'group' is an analysis of identical markers).

☒ All plots are contour plots with outliers or pseudocolor plots.

☒ A numerical value for number of cells or percentage (with statistics) is provided.

Methodology

Sample preparation	Venous blood samples were obtained from healthy volunteers, and buffy coats were purchased from the Welsh Blood Service. PBMCs were isolated via density gradient centrifugation using Histopaque-1077 (Sigma-Aldrich) and cryopreserved in fetal bovine serum containing 10% dimethyl sulfoxide (Sigma-Aldrich). Cells were cultured under standard conditions in RPMI 1640 medium supplemented with 10% fetal bovine serum, 100 U/mL penicillin, 100 ug/mL streptomycin, and 2 mM L-glutamine (all from Thermo Fisher Scientific) (R10).Cells were washed in Dulbecco's phosphate-buffered saline (Thermo Fisher Scientific), labeled for 15–30 min at room temperature with Zombie Aqua (BioLegend), and blocked for 10 min at 4 ºC with Human TruStain FcX (BioLegend). Surface stains were performed for 30 min at 4 ºC using combinations of the following directly conjugated monoclonal antibodies: (i) anti-CCR7–FITC (clone 150503) from BD Biosciences; (ii) anti-CD27–PC5 (clone 1A4CD27) from Beckman Coulter; (iii) anti-CCR7–BV421 (clone G043H7), anti-CD3–APC/Fire 750 (clone SK7), anti-CD3–PE-Cy5 (clone SK7), anti-CD3–PerCP (clone SK7), anti-CD4–BV605 (clone OKT4), anti-CD8a–BV711 (clone RPA-T8), anti-CD25–APC/Fire 750 (clone BC96), anti-CD45RA–FITC (clone HI100), anti-CD45RA–PE/Dazzle 594 (clone HI100), anti-CD69–APC (clone FN50), anti-CD69–BV421 (clone FN50), anti-CD69–BV785 (clone FN50), anti-CD69–FITC (clone FN50), anti-CD69–PE (clone FN50), anti-CD95–PE (clone DX2), anti-LAG-3–FITC (clone 11C3C65), anti-LAG-3–PE-Cy7 (clone 11C3C65), anti-PD-1–BV605 (clone EH12.2H7), anti-PD-1–PE (clone EH12.2H7), anti-PD-1–PE/Dazzle 594 (clone EH12.2H7), anti-TIGIT–BV421 (clone A15153G), anti-TIGIT–BV605 (clone A15153G), anti-TIM-3–BV785 (clone F38-2E2), anti-TIM-3–PE (clone F38-2E2), anti-TIM-3–PE/Dazzle 594 (clone F38-2E2), anti-TCR Vβ8–APC (clone JR2), and anti-TCR Vβ8–PE-Cy7 (clone JR2) from BioLegend; (iv) anti-TCR Vβ1–APC-Vio770 (clone REA662), anti-TCR Vβ2–FITC (clone REA654), and anti-TCR Vβ2–PE-Vio770 (clone REA654) from Miltenyi Biotec; and (v) anti-CD4–PE-Cy5.5 (clone S3.5) and anti-TCR Vβ12–FITC (clone S511) from Thermo Fisher Scientific. Cytosolic/intranuclear expression of Ki67 was detected using anti-Ki67–FITC (clone B56; BD Biosciences) in conjunction with a Foxp3 Transcription Factor Staining Buffer Kit (Thermo Fisher Scientific). Intracellular cytokines were exposed using a Cytofix/Cytoperm Plus Fixation/Permeabilization Solution Kit (BD Biosciences) and stained for 30 min at 4 ºC with combinations of the following directly conjugated monoclonal antibodies: (i) anti-IFN-g–APC (clone B27), anti-IFN-g–FITC (clone 4S.B3), anti-IL-2–PE/Dazzle 594 (clone MQ1-17H12), anti-TNF-a–BV605 (clone MAb11), and anti-TNF-a–BV785 (clone MAb11) from BioLegend; and (ii) anti-TNF-▯–APC-Vio770 (clone cA2) from Miltenyi Biotec. All flow cytometry panels were validated using individually stained Anti-Mouse IgK/Negative Control Particles (BD Biosciences). Data were acquired using a modified FACS Aria II (BD Biosciences) or an Attune NxT (Thermo Fisher Scientific) and analyzed using FlowJo software version 9.9.6 or version 10.7.1 (FlowJo LLC).
Instrument	BD FACSAria II (BD Biosciences); Attune NxT (ThermoFisher Scientific)
Software	FlowJo (v9.9.6 or 10.7.1; TreeStar)
Cell population abundance	No cell sorting was performed
Gating strategy	Lymphocytes were first gated (as determined on the basis of FSC SSC characteristics). Doublets were then excluded prior to gating live (live dead aqua viability dye negative) CD3+ cells. CD4+ and CD8+ T cells were then gated before selection of TRBV + cells (e.g. TRBV12-3/12-4+).

☒ Tick this box to confirm that a figure exemplifying the gating strategy is provided in the Supplementary Information.
